# Gene expression patterns associated with *Leishmania panamensis* infection in macrophages from BALB/c and C57BL/6 mice

**DOI:** 10.1371/journal.pntd.0009225

**Published:** 2021-02-22

**Authors:** Carlos M. Restrepo, Alejandro Llanes, Lizzi Herrera, Esteban Ellis, Ricardo Lleonart, Patricia L. Fernández

**Affiliations:** 1 Centro de Biología Celular y Molecular de Enfermedades, Instituto de Investigaciones Científicas y Servicios de Alta Tecnología (INDICASAT AIP), Panama City, Panama, Republic of Panama; 2 Departamento de Biotecnología, Facultad de Ciencias de la Salud, Universidad Latina de Panamá, Panama City, Panama, Republic of Panama; Universiteit Antwerpen, BELGIUM

## Abstract

*Leishmania* parasites can trigger different host immune responses that result in varying levels of disease severity. The C57BL/6 and BALB/c mouse strains are among the host models commonly used for characterizing the immunopathogenesis of *Leishmania* species and the possible antileishmanial effect of novel drug candidates. C57BL/6 mice tend to be resistant to *Leishmania* infections, whereas BALB/c mice display a susceptible phenotype. Studying species-specific interactions between *Leishmania* parasites and different host systems is a key step to characterize and validate these models for *in vivo* studies. Here, we use RNA-Seq and differential expression analysis to characterize the transcriptomic profiles of C57BL/6 and BALB/c peritoneal-derived macrophages in response to *Leishmania panamensis* infection. We observed differences between BALB/c and C57BL/6 macrophages regarding pathways associated with lysosomal degradation, arginine metabolism and the regulation of cell cycle. We also observed differences in the expression of chemokine and cytokine genes associated with regulation of immune responses. In conclusion, infection with *L*. *panamensis* induced an inflammatory gene expression pattern in C57BL/6 macrophages that is more consistently associated with a classic macrophage M1 activation, whereas in BALB/c macrophages a gene expression pattern consistent with an intermediate inflammatory response was observed.

## Author summary

Leishmaniasis is a disease caused by parasites of the *Leishmania* genus. *Leishmania panamensis* is a species that causes cutaneous leishmaniasis and potentially the more severe mucocutaneous leishmaniasis characterized by disfiguring lesions in nose and mouth. The C57BL/6 and BALB/c mouse strains are among the models commonly used for studying *Leishmania* infection and evaluating the effect of novel drug candidates. Previous research shows that the response of these two strains to *Leishmania* infection differs by factors that are related to both mouse strain genetic background and the infecting *Leishmania* species. In this study we characterized the differences in gene expression in macrophages of both mouse strains using next generation sequencing technologies. We identified differences in the expression of genes associated with the immune response that suggest that C57BL/6 macrophages display a classical pattern of activation associated with resolution of the disease, while BALB/c macrophages show an intermediate pattern of activation that favors parasite persistence and chronicity of the disease. The intermediate pattern observed in BALB/c macrophages resembles that observed in human infections with *L*. *panamensis* and supports the use of BALB/c as the preferred model for studying *L*. *panamensis* infection.

## Introduction

Kinetoplastid parasites of the *Leishmania* genus cause a diverse set of clinical presentations collectively known as leishmaniasis. *Leishmania* parasites exhibit a digenetic life cycle, with an extracellular promastigote form that lives in the sandfly vector and an intracellular amastigote that colonizes macrophages of mammalian hosts. Upon phagocytosis, *Leishmania* parasites develop within phagolysosome-derived structures known as parasitophorous vacuoles, where they delay cytotoxic responses in order to grow and replicate [1]. It has been consistently reported that *Leishmania* parasites can trigger different host immune responses that are responsible for the varying levels of disease severity [2,3]. The T helper cell 1 (Th1) cytokine response leads to a classical macrophage activation profile (M1), characterized by secretion of high levels of proinflammatory cytokines and production of reactive oxygen species (ROS) and nitrogen radicals. In contrast, the T helper cell 2 (Th2) cytokine response leads to an alternative macrophage activation profile (M2), characterized by inhibition of proinflammatory signals and subsequent increase in parasite number and disease exacerbation [4,5].

The C57BL/6 and BALB/c mouse strains are among the host model systems commonly used in studies characterizing the immunopathogenesis of *Leishmania* species and antileishmanial effect of novel drug candidates [2,6,7]. C57BL/6 mice show resistance to *Leishmania* infections, due to a predominant Th1 cytokine response and M1 macrophage activation [4,8–10]. Conversely, most *Leishmania* infections in BALB/c mice trigger an initial Th2 response conferring a susceptible phenotype that results in sustained parasite growth and tissue damage [2–4,11–14].

Gene expression studies using microarrays and RNA sequencing (RNA-Seq) have allowed a broader characterization of changes in gene expression occurring in mouse strains during infection with different *Leishmania* species, revealing differences in the strength of polarization towards each immune response profile [8–10,12–18]. Several studies have shown a generalized suppression of gene expression in BALB/c macrophages infected with *L*. *donovani* and *L*. *major* [12–14]. However, a different scenario has been reported for *L*. *chagasi* [15], with approximately 80% of genes not differentially expressed after infection. Studies conducted in BALB/c macrophages infected with *L*. *amazonensis* [16] or *L*. *major* [18] reported downregulation of genes encoding chemokine receptors and upregulation of genes involved in fatty acid biosynthesis. Strain-specific expression patterns, possibly associated with differences in pathogenesis, have been reported in C57BL/6 and BALB/c *L*. *major* infected macrophages [9]. Additionally, important changes in the global transcriptome profile of infected macrophages have been observed among different time points after infection [9,10]. These facts highlight the importance of studying species-specific interactions between *Leishmania* parasites and different host systems in order to characterize and validate these models for *in vivo* studies.

Pathogenic *Leishmania* species are divided into two main subgenera, *Leishmania* (*Leishmania*) and *Leishmania* (*Viannia*). Species belonging to the *L*. (*Viannia*) subgenus are exclusively present in Central and South America and cause mainly cutaneous leishmaniasis (CL), although they can migrate to the nasopharyngeal area and cause a more severe presentation called mucocutaneous leishmaniasis (MCL) [19–22]. Despite the extensive studies conducted with species from the *L*. (*Leishmania*) subgenus, to the best of our knowledge, no study has so far used differential expression analysis to characterize gene expression patterns in murine macrophages during infection with *L*. (*Viannia*) species. However, previous studies in murine models have reported more complex scenarios of cytokine production in response to these species, when compared to those observed for *L*. (*Leishmania*) [21,23–25]. In this study, we used RNA-Seq and differential expression analysis to characterize the transcriptomic profiles of C57BL/6 and BALB/c peritoneal-derived macrophages in response to *L*. *panamensis* infection. We also used functional enrichment analysis to identify and compare biological roles and pathways enriched by genes suspected to be differentially expressed in both mouse strains.

## Methods

### Ethics statement

All experimental procedures were approved by the Institutional Animal Care and Use Committee of INDICASAT (IACUC-14-002) and were based on the strict observance of the ethical guidelines related to the handling of laboratory animals, in accordance with international regulations and those established by INDICASAT.

### Mice

Female BALB/c and C57BL/6 mice, 8 weeks of age, were provided by INDICASAT’s animal facility. Animals were maintained with a 12 hours light/dark cycle, at a constant temperature of 24 °C with free access to food and water.

### Parasite culture

*Leishmania panamensis* PSC-1 strain (MHOM/PA/1994/PSC-1) promastigotes were axenically maintained in Schneider’s insect medium (Sigma-Aldrich, St. Louis, MO, USA) at pH 7.2 supplemented with 20% (v/v) heat-inactivated fetal bovine serum (Gibco, Gaithersburg, MD, USA), 50 μg/mL gentamicin (Sigma-Aldrich, St. Louis, MO, USA), and incubated at 25 °C. Parasites were previously adapted to mice by serial passages in the hind footpad of BALB/c mice for a period of 24 months [26].

### Murine macrophages isolation and infection assay

Peritoneal resident macrophages from BALB/c and C57BL/6 mice were collected by peritoneal lavage with 1X ice-cold phosphate-buffered saline (PBS) (Sigma-Aldrich, St. Louis, MO, USA). Cells were seeded in Roswell Park Memorial Institute medium (RPMI) (Gibco, Gaithersburg, MD, USA) supplemented with 10% (v/v) heat-inactivated fetal bovine serum, penicillin/streptomycin (100 U/mL/100 μg/mL) at a density of 1 x 10^6^ cells per well in 24-well plates with a round glass coverslip in each well and cultured for 2 h at 37 °C in an atmosphere of 5% CO_2_. Non-adherent cells were removed by washing with RPMI medium and adherent macrophages were infected with late stationary phase promastigotes at a parasite-to-cell ratio of approximately 30:1 (for TNF and IL-10 determination and RNA isolation) or 100:1 (for NO determination) and incubated for 4 h with the same incubation parameters. Non-internalized promastigotes were washed out and macrophages were incubated for 24 h at 37 °C. After incubation, supernatants were collected for further evaluation of secreted inflammatory mediators. Coverslips were washed once with PBS, fixed with methanol (Merck, Darmstadt, Germany), and stained with Giemsa (Sigma-Aldrich, St. Louis, MO, USA). The infection rate was calculated by counting the number of amastigotes per cell in a total of 250 cells.

For priming experiments, peritoneal macrophages from BALB/c mice were pre-treated for 2 h with 1 ng/mL of lipopolysaccharide (LPS) from *Escherichia coli* 0111:B4 (InvivoGen, San Diego, CA, USA). Then, cultures were washed with PBS and infected with *L*. *panamensis* with a parasite-to-cell ratio of 30:1. After 1h, the medium was replaced by RPMI supplemented with 10% FBS and penicillin/streptomycin (100 U/mL/100 μg/mL). Supernatants were harvested 24 h later for further analysis.

### Evaluation of inflammatory mediators

The concentrations of tumor necrosis factor (TNF) and interleukin 10 (IL-10) were measured in supernatants collected from infected and non-infected peritoneal macrophage cultures by using the DuoSet commercial ELISA kit (R&D System, USA), following manufacturer’s instructions. For nitric oxide (NO) determination, nitrite levels were determined as an indicator of NO production using the Griess Reagent System (Promega, Madison, WI, USA) according to manufacturer’s protocol. Statistical analyses were performed with GraphPad Prism version 6.00, (GraphPad Inc., La Jolla, CA). Statistical differences between groups were evaluated by Student’s t-test. A difference between groups was considered to be significant if *P* < 0.05.

### RNA isolation and transcriptome sequencing

*Leishmania*-infected and non-infected peritoneal macrophages, as described above, were processed independently for each mouse strain. The experiment was performed in triplicate. Macrophages were lysed directly in 1 mL of Trizol (Invitrogen, Carlsbad, CA, USA) and total RNA was purified using chloroform separation and isopropanol precipitation. Integrity and presence of contaminants in the RNA samples were assessed using the Agilent RNA 6000 Nano kit on a 2100 Bioanalyzer system (Agilent Technologies, Santa Clara, CA, USA). RNA concentration was estimated by the Picogreen (Invitrogen, Carlsbad, CA, USA) method using Victor 3 fluorometry (PerkinElmer, Waltham, MA, USA). RNA was stored at -80 °C until used for sequencing. Libraries of complementary DNA (cDNA) fragments were generated from polyadenylated (poly(A)) RNA using the TruSeq RNA v2 sample preparation kit (Illumina, San Diego, CA, USA). Fragment libraries were sequenced in a NovaSeq 6000 instrument (Illumina, San Diego, CA, USA), for a total throughput of 100 million 150-bp paired-end reads per sample.

### Read mapping and abundance estimation

HISAT2 (version 2.1.0) [27] was used to align the cDNA-derived reads from uninfected and *L*. *panamensis*-infected macrophages to the *L*. *panamensis* and mouse reference genomes, using the default parameters for paired-end and non-strand-specific RNA-Seq data. The *L*. *panamensis* reference genome was downloaded from the Genbank database, under BioProject PRJNA235344 [28]. The mouse reference genome (mm10, build name GRCm38) was obtained from the UCSC Genome Browser (http://genome.ucsc.edu/). Transcript abundance was estimated from read alignments by using FeatureCounts (version 1.6.3) [29]. Suspected non-expressed genes, defined as gene features having counts equal to zero or only a single count across all samples, were removed prior to subsequent analyses.

### Data quality assessment and differential expression analysis

In order to visualize the relationships between samples, counts were first normalized correcting for differences in sequencing depth and subsequently log_2_ transformed. Variance across the mean was stabilized using the variance stabilizing transformation (VST) algorithm, as suggested by Anders et al. [30] for negative binomial data with a dispersion-mean trend. Subsequently, Euclidean distances between samples were calculated from the transformed data and the relationships between samples were visualized by using principal component analysis (PCA). Additionally, the Pearson correlation coefficient was calculated from the transformed counts to assess the similarity of RNA-Seq samples in a pairwise fashion and results were visualized as a heat map using the complement of the correlation coefficient as a measurement of distance.

Differential expression analysis was conducted on raw counts using the DESeq2 R package (version 1.12.3) [31]. Briefly, the DESeq2 algorithm normalizes for differences in sequencing depth among samples, estimates dispersion values for each gene and fits a generalized linear model to the data. The distributions of estimated coefficients in the model for each mouse strain were visualized using *log* ratio and mean average plots (MA-plots). Genes with a Benjamini-Hochberg (BH) multiple-testing adjusted *P* value of < 0.05 were defined as differentially expressed. Only those genes with a logarithm to the base 2 of fold change (*log*_2_FC) of at least 0.5 were considered for subsequent functional enrichment analysis.

### Functional enrichment analysis

Gene ontology (GO) enrichment analyses were performed with the GO_Slim Chart Tool and the GO Term Mapper provided by the Mouse Genome Informatics (MGI) resource (http://www.informatics.jax.org) and based on the Mouse Genome Database [32]. REVIGO [33] was further used to simplify the list of enriched terms obtained with GO Term Mapper, by discarding terms with a false discovery rate (FDR) above 0.5 and a dispensability value above 0.7.

Identification of signaling and metabolic pathways overrepresented in the murine differentially expressed genes was performed by using the enrichKEGG function implemented in the ClusterProfile R package (version 3.12.0) [34] for pathways defined in the Kyoto Encyclopedia of Genes and Genomes (KEGG) database. The enrichKEGG function implements a hypergeometric test for each KEGG pathway and returns a *P* value. A cutoff of 0.05 was used to identify enriched KEGG pathways. In order to detect variations specific to each mouse strain, two independent lists of up- and downregulated genes per strain were separately used as input files.

## Results

### RNA sequencing and data quality assessment

In order to characterize the global transcriptional response to infection, RNA-Seq and differential expression analysis were made in macrophages from the BALB/c and C57BL/6 mouse strains infected with *L*. *panamensis*. The percentages of infected macrophages were 99.5 ± 0.5% for BALB/c and 62.1 ± 0.3% for C57BL/6. Poly(A)-enriched RNA was isolated from uninfected and infected samples at 24 hours post-infection. Sequencing with the Illumina platform resulted in ~120 million paired-end 150-bp sequence reads per sample. Sequence data generated in this study was submitted to the Sequence Read Archive (SRA) and is accessible through BioProject PRJNA656921. On average, 98% of reads from uninfected samples and 90% of those from infected samples mapped unambiguously to the mouse reference genome. As expected, less than 0.01% of reads from the uninfected samples mapped against the *L*. *panamensis* reference genome. However, only 8–9% of the reads from infected samples mapped unambiguously to this reference genome, suggesting an overrepresentation of murine RNA in infected samples with respect to *Leishmania* RNA. Due to the relatively low coverage obtained after mapping the reads from infected samples against the *L*. *panamensis* reference genome (0–4 reads per protein-coding gene), we were not able to perform differential expression analysis for *Leishmania* genes.

**Fig 1:**
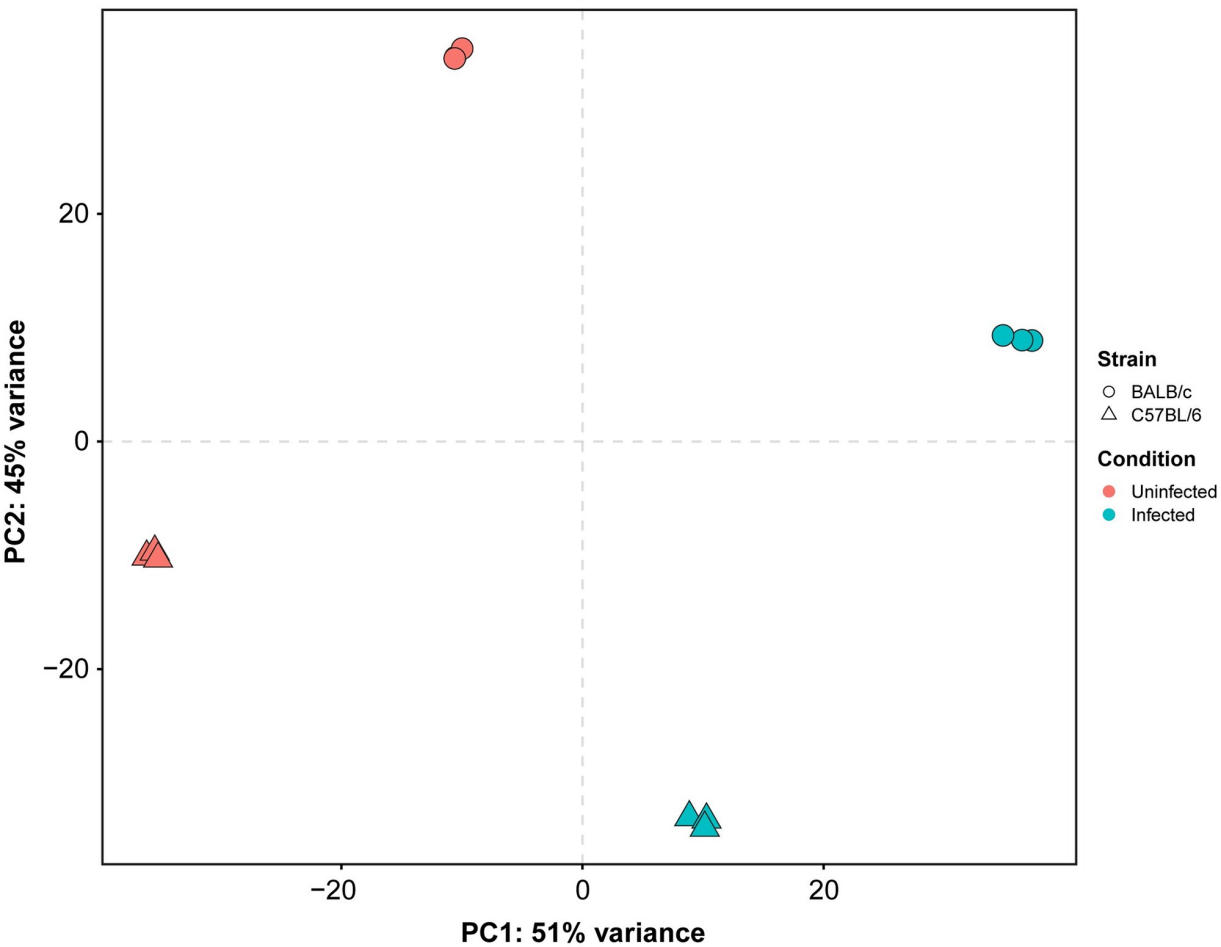
Principal component analysis (PCA) plot based on Euclidean distances of RNA-Seq counts. The two-dimensional diagram helps visualize clusters of correlated samples. The first (PC1; *x* axis) and second (PC2; *y* axis) principal components explain 96% of the total variance and show that samples properly cluster together according to infection condition (uninfected or infected) and mouse strain (BALB/c or C57BL/6).

A preliminary principal component analysis (PCA) was performed to visualize clustering patterns in our samples. PCA works best for homoscedastic datasets, in which the expected amount of variance is approximately the same across different mean values [30], however, in RNA-Seq counts, the expected variance increases with the mean (S1 Fig). In order to avoid the overestimation of sample distances that can result from using raw RNA-Seq counts, data was first transformed by using the variance stabilizing transformation (VST) algorithm [30], which performs a modified *log*_2_ transformation that corrects the variance overestimation resulting from taking the logarithm of small counts. Scatter plots of transformed counts of biological replicates show that variance across low- and high-count genes was notably compressed (S2 Fig). The PCA plot based on Euclidean distances of our transformed data (Fig 1) was able to explain 96% of the total variance and showed that samples properly cluster together when considering infection and mouse strain as different conditions, thus fitting the expectations of our experimental design. Infected and uninfected samples were respectively located towards the right and left ends of the first principal component (PC1; *x* axis), whereas BALB/c and C57BL/6 were located towards the upper and lower ends of the second principal component (PC2; *y* axis). Comparison of normalized counts also revealed good correlation among biological replicates for each infection condition and mouse strain, with a Pearson correlation coefficient greater than 0.9 in all cases (S3 Fig).

### Differential expression analysis

**Fig 2:**
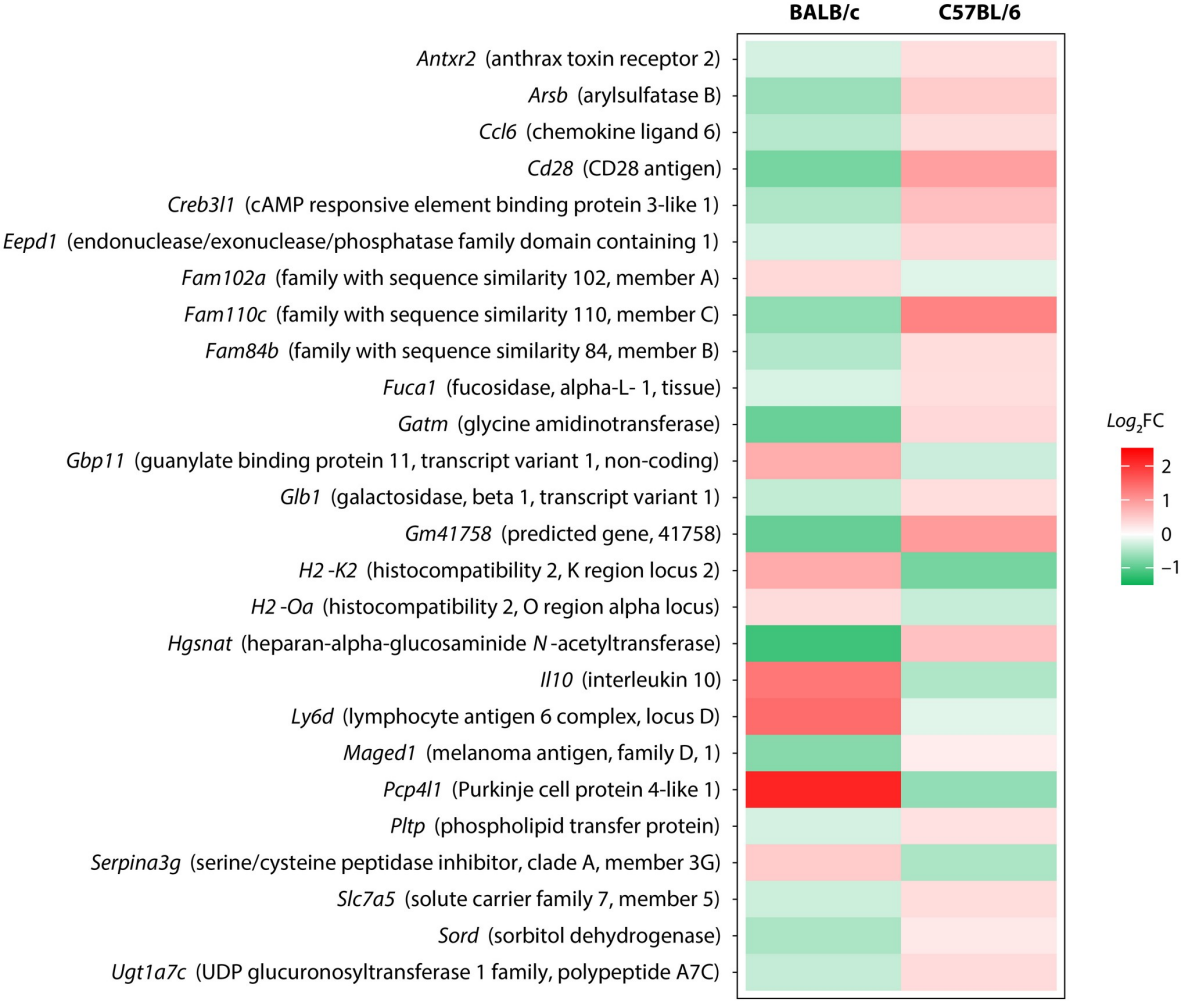
Genes suspected to be reciprocally regulated in BALB/c and C57BL/6 macrophages infected with *L*. *panamensis*. Color scale represents the binary logarithm of expression fold change between uninfected and infected macrophages from each strain (*log*_2_FC). Red indicates upregulation (*log*_2_FC > 0) and green indicates downregulation (*log*_2_FC < 0). cAMP: cyclic adenosine monophosphate, CD28: cluster of differentiation 28, UDP: uridine diphosphate.

Differential expression analysis revealed a relatively high number of genes whose transcript levels changed between conditions in both mouse strains (S4 Fig). Globally, we found 3,577 differentially expressed (DE) genes in BALB/c macrophages (2,107 up- and 1,470 downregulated) (S1 Table) and 4,761 DE genes in C57BL/6 macrophages (2,765 up- and 1,996 downregulated) (S2 Table), considering only those genes with a BH multiple-testing adjusted *P* value of < 0.05. Of all these DE genes, 3,023 were found to be differentially expressed in the macrophages of both BALB/c and C57BL/6 mice. Only 26 of these genes were differentially expressed in opposite directions when comparing the expression patterns of the two mouse strains (Fig 2), suggesting that their expression may be reciprocally regulated in these strains. Conversely, 554 DE genes (210 up- and 344 downregulated) and 1,735 DE genes (855 up- and 880 downregulated) were specific to BALB/c (S3 Table) and C57BL/6 (S4 Table) macrophages, respectively.

### Functional enrichment analysis

We used gene ontology (GO) and pathway enrichment analysis to explore biological functions and processes. GO enrichment analysis was used to explore the GO terms overrepresented in DE genes. First, we used GO Slim categories to explore enrichment in the relatively large sets of genes suspected to be exclusively upregulated in BALB/c or C57BL/6. GO Slims are reduced versions of gene ontologies, containing a subset of representative GO terms, that provide a global view of enriched biological functions, while avoiding unnecessarily long lists of specific GO terms. Almost all GO Slim categories were found to be similarly enriched by exclusively upregulated genes in both mouse strains (S5 Fig).

**Fig 3:**
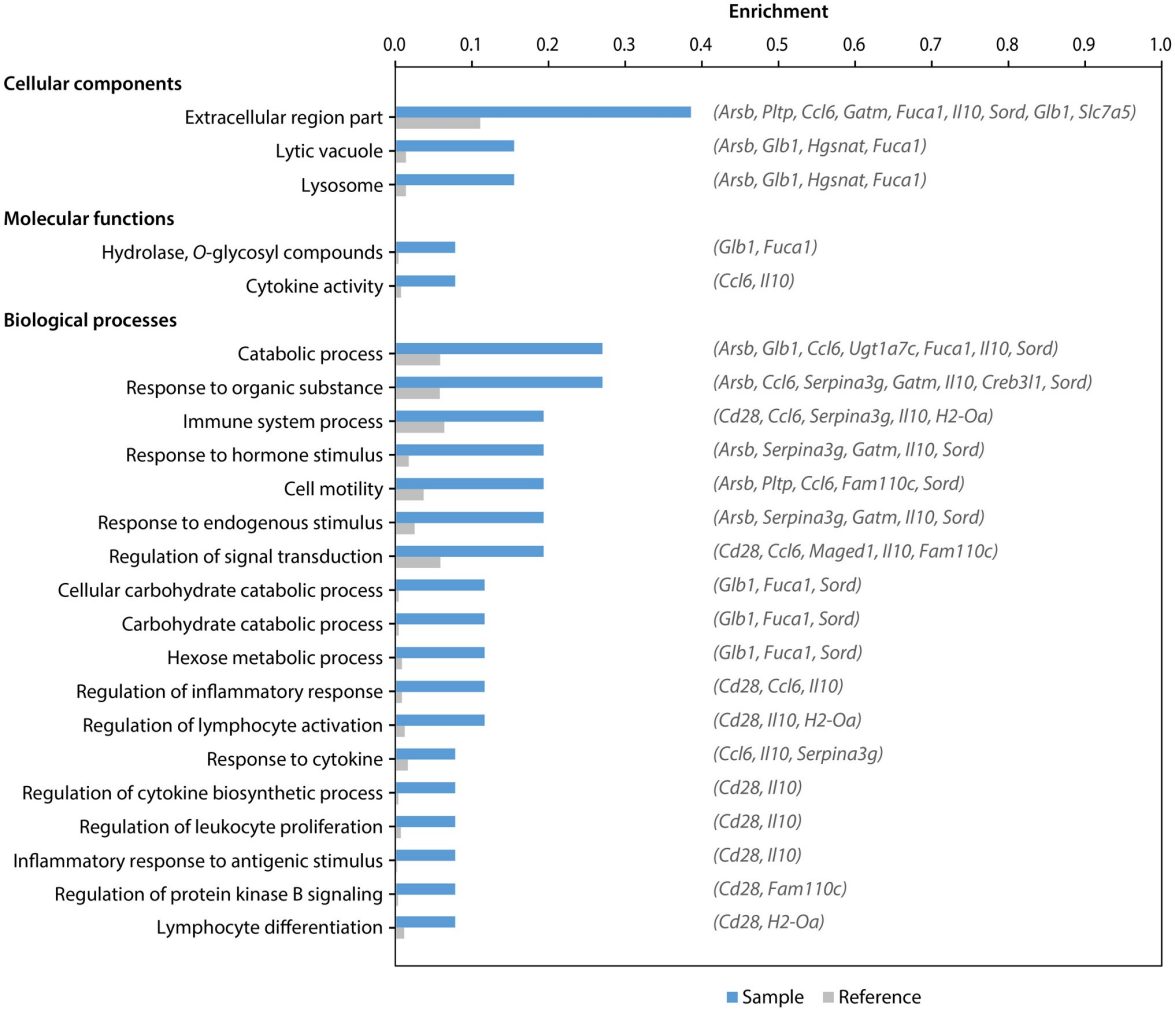
Gene ontology (GO) terms overrepresented in DE genes suspected to be reciprocally regulated in infected BALB/c and C57BL/6 macrophages. Figure shows representative GO terms (FDR < 0.5) inferred from the 26 genes found to be differentially expressed in opposite directions (sample), when compared to those from all the annotated genes in the mouse genome (reference). Symbols for genes associated with each GO term are listed alongside the corresponding sample bars. Gene products are shown in Fig 2.

To further explore the functional differences between the two mouse strains, we then performed GO enrichment analysis with entire gene ontologies and the set of 26 genes suspected to be reciprocally up- or downregulated in each strain. This analysis resulted in a list of 211 enriched GO terms, which was further reduced to a non-redundant set of representative terms by using REVIGO (S5 Table). Results revealed significant enrichment of terms associated with degradation, lysosome and lysosome-derived lytic vacuoles, as well as several immune system processes (Fig 3), suggestive of possible differences between both strains in these biological categories. For instance, genes encoding an arylsulfatase (*Arsb*), an α-L-1 fucosidase (*Fuca1*), an heparan-specific *N*-acetyltransferase (*Hgsnat*) and a β-galactosidase (*Glb1*), all associated with lysosomal degradation, appear to be downregulated in BALB/c but upregulated in C57BL/6. Among the genes associated with the immune system, those encoding chemokine CCL6 (*Ccl6*) and cluster of differentiation (CD) 28 (*Cd28*) seem to be downregulated in BALB/c but upregulated in C57BL/6, whereas those encoding the α subunit of major histocompatibility complex (MHC) class II molecules (*H2-Oa*) and IL-10 (*Il10*) are upregulated in BALB/c.

To further identify known cellular processes within the differential expression profiles of each mouse strain, we also performed a pathway enrichment analysis to find pathways from the KEGG database enriched by up- or downregulated genes in both mouse strains. In BALB/c, 75 KEGG pathways were found to be enriched by 717 DE genes, 56 and 19 when considering up- and downregulated genes, respectively (S6 Table). Conversely, 122 KEGG pathways were found to be enriched by 1,079 DE genes in C57BL/6, 67 and 55 when considering up- and downregulated genes, respectively (S7 Table). Pathways found to be enriched by upregulated genes in both mouse strains (S8 Table) were mainly associated with central metabolic routes, including glycolysis, gluconeogenesis, citric acid cycle, 2-oxocarboxylic acid metabolism, oxidative phosphorylation, fatty acid elongation, cholesterol metabolism and biosynthesis of amino acids. Additionally, pathways related to phagocytosis, processing of intracellular parasites and immune response were also found to be enriched by upregulated genes, including those involving the phagosome, proteasome, lysosome, as well as antigen processing and presentation pathways, glutathione metabolism and the nucleotide-binding oligomerization domain-like (NOD-like) receptor signaling pathway. Pathways enriched by downregulated genes in both mouse strains (S8 Table) comprise mostly those related to immune and inflammatory responses, including Th1 and Th2 cell differentiation, the signaling pathways of nuclear factor κ-light-chain-enhancer of activated B cells (NF-κB), FoxO, tumor necrosis factor (TNF) and chemokines, as well as the complement and coagulation cascades. Disease-specific KEGG pathways such as Leishmaniasis (KEGG accession mmu05140), Toxoplasmosis (mmu05145) and Chagas disease (mmu05142), which represent comprehensive assemblies of observations from multiple studies, were also largely enriched by shared genes involved in response to infection.

Interestingly, only four pathways were found to be exclusively enriched by DE genes in BALB/c (Table 1), all of which were enriched by upregulated genes. One such pathway is the regulation of the cell cycle, which was found to be enriched by genes encoding growth arrest and DNA-damage-inducible (GADD) proteins such as GADD45A (*Gadd45a*), GADD45B (*Gadd45b*), GADD45G (*Gadd45g*) and GADD45GIP1 (*Gadd45gip1*). These genes were upregulated in macrophages of both mouse strains, but the fold change of *Gadd45g* was 1.5 times higher in BALB/c. However, the *Gadd45a* gene was downregulated in BALB/c but its expression was not significantly altered in C57BL/6. Furthermore, the apoptosis-activating serine protease granzyme B gene (*Gzmb*) was overexpressed in both strains, but its fold change was 2.4 times higher in C57BL/6.

**Table 1:** KEGG pathways exclusively enriched by DE genes in infected BALB/c macrophages.

Accession number	KEGG Pathway	DE genes	Pathway size	Adjusted *P* value
Enriched by upregulated genes				
mmu04110	Cell cycle	25	123	4.81E-03
mmu05166	Human T-cell leukemia virus 1 infection	41	246	8.47E-03
mmu05416	Viral myocarditis	19	88	8.99E-03
mmu05320	Autoimmune thyroid disease	17	78	1.32E-02

Conversely, we found 51 pathways exclusively enriched by DE genes in C57BL/6, many of which are associated with infection and immune system functions (Table 2 and S9 Table). Among these, the TNF signaling pathway, arginine and proline metabolism and the IL-17 signaling pathway were enriched by upregulated genes. Notably, the majority of genes involved in arginine and proline metabolism, including those encoding arginase 1 (*arg1*), were upregulated to a greater extent in C57BL/6, while the gene encoding the cationic amino acid transporter (CAT) member 2 (*Slc7a2*) was found to be upregulated in BALB/c macrophages. Pathways exclusively enriched by downregulated genes in C57BL/6 include those related to infection with specific intracellular pathogens, Th17 cell differentiation, cytokine-cytokine receptor interaction and signaling pathways of B-cell, T-cell and Toll-like receptors (TLRs). These pathways were mostly enriched by genes shared among them, including those encoding IL-1β (*Il1b*), IL-10 (*Il10*), transforming growth factor (TGF) β (*Tgfb3*), CXCL9 (*Cxcl9*) and CCR2 (*Ccr2*). These genes are also shared with other immune-related pathways mentioned above, including the TNF signaling pathway.

**Table 2:** KEGG pathways exclusively enriched by DE genes in infected C57BL/6 macrophages.

Accession number ^1^	KEGG Pathway	DE genes	Pathway size	Adjusted *P* value
Enriched by upregulated genes				
mmu04668	TNF signaling pathway	31	113	5.44E-04
mmu00330	Arginine and proline metabolism	17	53	3.07E-03
mmu04657	IL-17 signaling pathway	21	91	4.07E-02
Enriched by downregulated genes				
mmu04659	Th17 cell differentiation	20	102	3.29E-04
mmu05168	Herpes simplex virus 1 infection	53	437	3.65E-04
mmu04662	B cell receptor signaling pathway	17	81	4.54E-04
mmu05143	African trypanosomiasis	11	38	5.36E-04
mmu05144	Malaria	13	56	1.03E-03
mmu05161	Hepatitis B	25	162	1.10E-03
mmu05169	Epstein-Barr virus infection	31	228	1.60E-03
mmu05164	Influenza A	24	166	3.36E-03
mmu04660	T cell receptor signaling pathway	17	103	4.62E-03
mmu05150	Staphylococcus aureus infection	19	122	4.62E-03
mmu05166	Human T-cell leukemia virus 1 infection	31	246	4.62E-03
mmu05162	Measles	21	144	5.49E-03
mmu04620	Toll-like receptor signaling pathway	16	99	7.67E-03
mmu04145	Phagosome	23	180	1.67E-02
mmu05134	Legionellosis	10	61	3.75E-02
mmu04060	Cytokine-cytokine receptor interaction	31	295	4.39E-02

^1^ This table only shows pathways potentially related to infection and immune system functions. Full list of pathways exclusively enriched by DE genes in infected C57BL/6 macrophages is shown in S9 Table.

**Fig 4:**
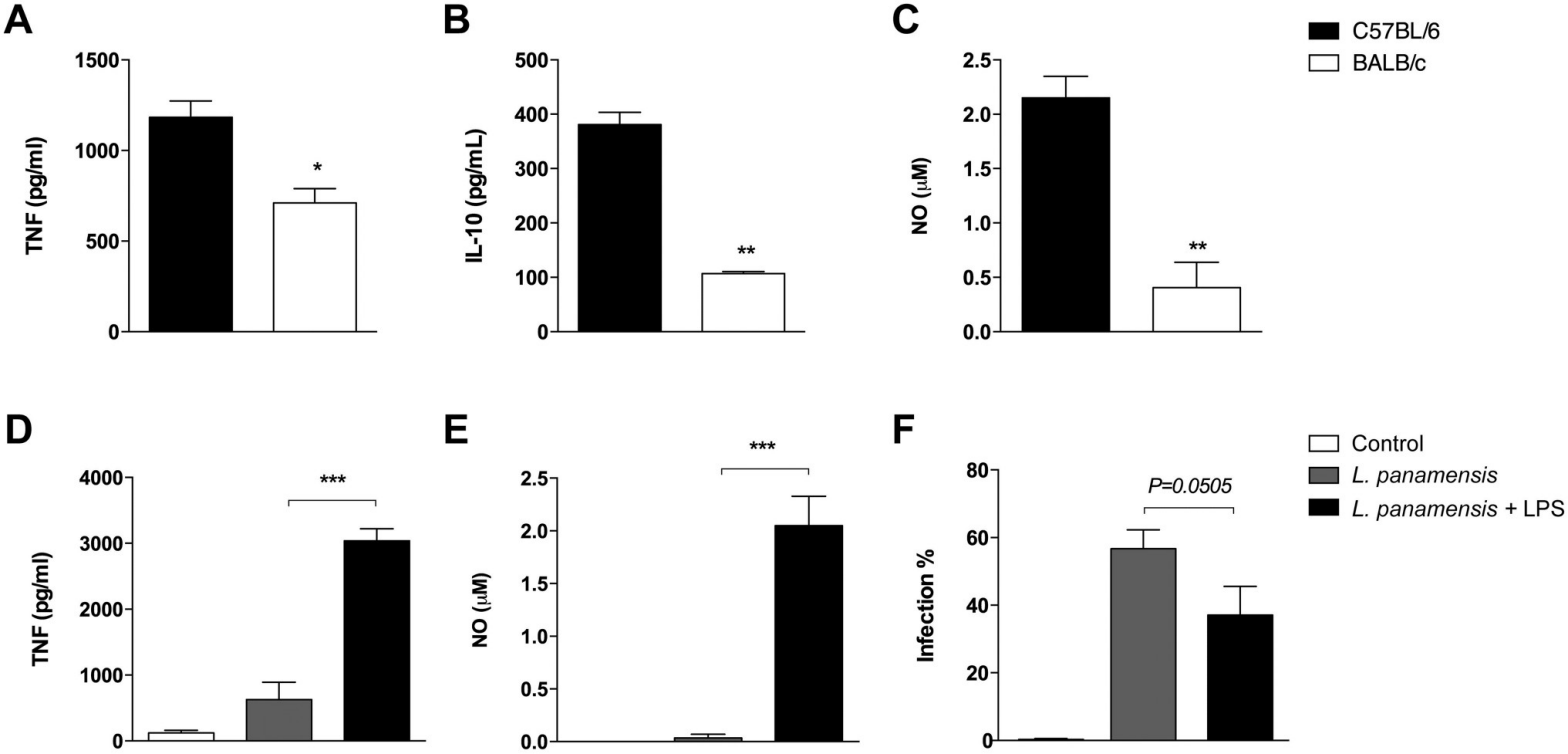
Production of inflammatory mediators by infected macrophages. Peritoneal macrophages (1 × 10^6^) were infected with *L*. *panamensis* in a parasite to cell ratio of 30:1 **(A-B)** or 100:1 **(C)**. Production of TNF **(A)**, IL-10 **(B)** and NO **(C)** was determined in the supernatants of infected cells after 24 hours of infection. **(D-F)** Peritoneal macrophages from BALB/c mice were primed with LPS (1 ng/mL) for 2 h and then infected with *L*. *panamensis* in a parasite-to-cell ratio of 30:1. Supernatants were collected after 24 h for TNF **(D)** and NO **(E)** quantification. Percentage of infection was also included in the figure **(F)**. Data represent mean ± SEM from stimuli performed in duplicates of two independent experiments. * *P* < 0.05; **, *P* < 0.01; ***, *P* < 0.001.

Since the TNF signaling pathway was distinctively enriched by DE genes in both strains, we measured the production of TNF by infected macrophages. Results showed significantly higher levels of TNF in C57BL/6 when compared to BALB/c (Fig 4A). This finding is consistent with the relatively high number of DE genes enriching the TNF signaling pathway, which encode several cytokines, chemokines and mitogen-activated protein kinases (MAPK). Among the cytokine genes whose expression was altered by *L*. *panamensis* infection were those encoding proinflammatory cytokine IL-1β (*Il1b*) and anti-inflammatory M2-polarizing cytokines IL-4 (*Il4*), IL-10 (*Il10*) and TGFβ (*Tgfb3*). TGFβ was downregulated in both strains, IL-10 was upregulated in BALB/c and downregulated in C57BL/6, IL-4 was exclusively downregulated in BALB/c and IL-1β was exclusively downregulated in C57BL/6.

Since both the gene ontology and pathway enrichment analysis suggested that IL-10 is reciprocally regulated in both mouse strains, we measured production of this cytokine by infected macrophages. Surprisingly, the levels of this protein were found to be higher in C57BL/6, instead of BALB/c (Fig 4B). However, C57BL/6 macrophages produced significantly higher levels of nitric oxide (NO) than BALB/c macrophages in response to *L*. *panamensis* infection, consistent with the M1 and M2 phenotypes, respectively (Fig 4C).

Infection with *L*. *panamensis* also seemed to modulate the expression of several chemokines related to the TNF pathway, including the monocyte chemoattractant protein (MCP) 1/CCL2 (*Ccl2*), macrophage inflammatory proteins (MIP)-1α/CCL3 (*Ccl3*) and -1β/CCL4 (*Ccl4*), CCL12 (*Ccl12*), CXCL1 (*Cxcl1*), CXCL2 (*Cxcl2*), the interferon-γ-inducible protein (IP)-10/CXCL10 (*Cxcl10*), CXCL5 (*Cxcl5*) and the RANTES protein/CCL5 (*Ccl5*). Nonetheless, the expression of these chemokine genes was not modulated to the same extent in both mouse strains. The expression of *Cxcl1*, *Cxcl2* and *Cxcl5* was upregulated in C57BL/6 but not in BALB/c. Moreover, chemokine genes *Ccl3* and *Ccl4* were upregulated in both mouse strains but their fold change was 1.5 times higher in C57BL/6. Conversely, *Ccl2*, *Cxcl3* and *Cxcl10* were upregulated roughly at the same extent in both strains, whereas *Ccl5* and *Ccl12* had fold changes five times higher in BALB/c than in C57BL/6. Notably, even though *Ccl2* was upregulated in BALB/c and C57BL/6, its receptor CCR2 (*Ccr2*) was downregulated in both strains. Interestingly, the gene encoding the Th1-attracting protein CXCL9 (*Cxcl9*) was apparently downregulated by *L*. *panamensis* in both strains.

Additionally, expression of certain MAPK genes associated with the TNF pathway was altered, particularly those known for their role in regulating the induction of inflammatory cytokines and apoptotic signals. Genes encoding MAP2K3 (*Map2k3*) and MAP2K1 (*Map2k1*) were upregulated in both mouse strains. Moreover, expression of genes encoding MAPK11/p38 MAPK-β (*Mapk11*), MAP3K5/Apoptosis signal-regulating kinase (ASK) 1 (*Map3k5*) and MAPK3/Extracellular signal-regulated kinase (ERK) 1 (*Mapk3*) were only significantly altered by *L*. *panamensis* infection in C57BL/6 mice, with the first two genes downregulated and the latter, upregulated. It has been observed that IFN-γ priming alters the pattern of MAPKs expression in response to *L*. *donovani* infection, promoting leishmanicidal activity [35]. Low doses of lipopolysaccharide (LPS) prime the expression of pro-inflammatory mediators preparing cells for later challenges [36]. We observed that LPS priming of BALB/c macrophages promotes an M1 characteristic response with the secretion of high levels of TNF and NO (Fig 4D and 4E). This response was translated into a higher capacity of these cells to reduce the infection (Fig 4F).

## Discussion

*Leishmania* parasites are intracellular organisms known for their ability to efficiently colonize macrophages by regulating phagolysosome maturation, causing a delay in cytotoxic responses that allows them to grow and replicate. C57BL/6 mice often display a resistant phenotype to infection with most *Leishmania* species, due to a predominant proinflammatory macrophage activation profile (M1), often resulting in parasite clearance [4,8–10]. Conversely, BALB/c mice tend to exhibit a susceptible phenotype, possibly associated with initial alternative macrophage activation profile (M2), characterized by inhibition of proinflammatory signals and leading to sustained parasite growth and tissue damage [2–4,11–14]. However, there is conflicting evidence regarding the direction of polarization of macrophage activation, based on studies considering different combinations of *Leishmania* species and host mouse strains, which highlights the importance of studying species-specific interactions between *Leishmania* parasites and different host systems [8,9,23,10,12–18]. Here, we compared the transcriptomic profiles of C57BL/6 and BALB/c peritoneal-derived macrophages in response to *Leishmania panamensis* infection. Our results show both shared and distinct features in the transcriptomic profiles of C57BL/6 and BALB/c macrophages after infection with *L*. *panamensis* (S6 Fig). Several of the DE genes observed in the transcriptomic signatures for both mouse strains have been previously reported as differentially expressed in studies conducted with other *Leishmania* species [8–10,14–16,18], although the direction and intensity of fold change varies. Similarities and differences concerning DE genes reported in this study for *L*. *panamensis* infection are discussed.

In both mouse strains, we observed an overall induction of central metabolic pathways mostly related to energy, lipid and cholesterol metabolism. This induction of central metabolic pathways seems to be a common hallmark of *Leishmania* infection, since similar findings have been reported in previous studies with *L*. *major* and *L*. *amazonensis* [8–10,16,18,37]. Authors have suggested that *Leishmania* parasites push macrophages towards anaerobic glycolysis and promote lipid and cholesterol accumulation in the parasitophorous vacuoles and their proximities [18]. Accumulated lipids are thought to promote recruitment and retention of key proteins to the parasitophorous vacuoles, in addition to their role as nutrients.

Functional enrichment analysis of exclusively upregulated genes performed with global GO Slim categories showed roughly similar enrichment values for both mouse strains. However, GO enrichment analysis of DE genes suspected to be reciprocally regulated in both mouse strains revealed significant enrichment of several biological roles. These findings support the previously mentioned reports of a global induction of central cellular processes in both mouse strains after infection with *Leishmania*, with only particular differences in certain biological processes. Among the GO terms and pathways that were found to be enriched by DE genes are those associated with lysosomal degradation and lysosome-derived lytic vacuoles, in some cases suggesting differences between BALB/c and C57BL/6 macrophages. This is not surprising, since the parasitophorous vacuoles in which *Leishmania* parasite grow are similar to phagolysosomes and their origin is associated with the endocytic pathway [1]. At least four genes associated with lysosomal degradation appear to be downregulated in BALB/c macrophages but upregulated in those from C57BL/6, which may contribute to the susceptible and resistant phenotypes respectively associated with these mouse strains. Conversely, most genes encoding lysosomal proteases were upregulated in both mouse strains, although the fold changes of these genes were higher in C57BL/6 than in BALB/c macrophages. Additionally, the gene encoding an important lysosomal protease, cathepsin D (*Ctsd*), was exclusively upregulated in C57BL/6. These findings differ from those reported in previous studies with *L*. *major* [9,18], in that most genes encoding lysosomal proteases were found to be downregulated by *Leishmania* infection. Our results suggest that, despite differences in susceptibility to infection between both mouse strains, modulation of the expression of genes encoding lysosomal enzymes may also vary among *Leishmania* species, thus potentially compromising survival of the parasites within lytic vacuoles.

We also observed several differences between BALB/c and C57BL/6 macrophages regarding the expression of chemokine, MAPK and cytokine genes shared by several KEGG pathways associated with immune system functions, remarkably, the TNF signaling pathway. C57BL/6 macrophages displayed a consistent induction of chemokine genes *Ccl2*, *Ccl3*, *Ccl4*, *Ccl5*, *Ccl6*, *Ccl12*, *Cxcl1*, *Cxcl2*, *Cxcl3*, *Cxcl5* and *Cxcl10*, which define a strong and redundant cascade of inflammatory signals for recruitment of monocytes and neutrophils, characteristic of a M1 macrophage activation profile [4,5]. In contrast, several of these chemokine genes showed lower fold changes or no change at all in BALB/c macrophages, suggesting their activation profile could be closer to M2 or intermediate between the M1 and M2 profiles. Notably, genes encoding the Th1-attracting chemokine CXCL9 (*Cxcl9*) and the CCL2 receptor, CCR2 (*Ccr2*), were found to be downregulated in both mouse strains after infection, but this downregulation was greater in BALB/c macrophages. Previous studies in BALB/c and C57BL/6 macrophages challenged with *L*. *major* have shown a similar downregulation of *Ccr2*, although in these studies, *Ccl2* remained upregulated even in BALB/c [9,18].

Although various MAPK genes known for regulating the activation of inflammatory cytokines and apoptotic signals were upregulated in both mouse strains, expression of genes encoding key regulators MAPK11/p38 MAPK-β (*Mapk11*) and MAP3K5/ASK1 (*Map3k5*) was not increased after infection in BALB/c, further supporting the occurrence of an intermediate M2-like profile in this strain. MAPK11/p38-MAPK-β functions are mostly redundant with those of MAPK14/p38-MAPK-α, playing an important role in the cascades of cellular responses to extracellular stimuli and leading to direct activation of transcription factors [38]. MAP3K5/ASK1 is activated in response to various cytotoxic stresses and is required for TNF- and oxidative stress-induced sustained activation of p38 MAPKs and apoptosis [39]. Activation of p38-MAPKs is associated with phagosome maturation and induction of inflammatory mediators in *Leishmania*-infected murine macrophages [40,41], thus a reduction in the activation of this kinase might affect the production of chemokines described above. Collectively, the expression pattern of the chemokine and kinase genes observed in our study may imply a reduced activation of the TNF signaling pathway in BALB/c macrophages, consistent with lower protein levels of TNF observed in BALB/c macrophages compared to those from C57BL/6.

M2 macrophages are characterized by the active production of the anti-inflammatory cytokine IL-10 [4,5,42], whose gene (*Il10*) expression was upregulated in BALB/c and downregulated in C57BL/6, further supporting an M2-like activation in BALB/c. Surprisingly, protein levels of IL-10 at 24 h post-infection were significantly higher in C57BL/6 than BALB/c macrophages. This observation could be the result of the kinetics of cytokine production induced by *L*. *panamensis*, as upregulation of this cytokine could have occurred in macrophages of both mouse strains earlier in the infection [10,37]. However, as infection progresses, C57BL/6 macrophages could have been able to downregulate *Il10* expression, shifting towards an M1 phenotype. Although alternative M2 activation has been observed in macrophages exposed to IL-4, IL-13, TGFβ and glucocorticoids [42], production of IL-4, TGFβ and autocrine regulation by the macrophage seem to be dispensable for M2 activation [42,43]. Therefore, the downregulation of IL-4 (*Il4*) and no significant change in TGFβ (*Tgfb3*) transcript levels observed in BALB/c macrophages may not have affected their transition to an M2-like phenotype.

Additional findings associated with arginine metabolism and the regulation of cell cycle also suggest two distinct expression patterns in C57BL/6 and BALB/c macrophages. For instance, the fold change of arginase 1 (*Arg1*), an enzyme that competes with nitric oxide synthase for its substrate, arginine, was higher in C57BL/6 than BALB/c macrophages. Induction of arginase causes arginine depletion, which leads to suppressed T cell and NK cell proliferation and proinflammatory cytokine secretion, promoting shifting of macrophages to an alternative M2 activation [5,44]. However, transcript levels of the cationic amino acid transporter CAT2, a protein involved in arginine uptake, were only increased in BALB/c macrophages. Relatively low expression of the gene encoding this transporter (*Slc7a2*) has also been reported in C57BL/6, possibly due to a deletion of an AGGG repeat on its promoter [45]. The lower expression of this transporter is thought to reduce the effects of arginase 1 overexpression in C57BL/6, possibly contributing to a M1 profile in this mouse strain [45]. Additionally, several genes involved in regulation of cell cycle and apoptosis were upregulated in both mouse strains, in agreement with previous reports of generalized activation of pro-apoptotic signals in response to intracellular parasite-induced cytotoxic stress [8–10,46]. However, the gene encoding growth arrest and DNA-damage-inducible protein GADD45A (*Gadd45a*) was downregulated in BALB/c, whereas the pro-apoptotic granzyme B gene (*Gzmb*) showed a heightened expression in C57BL/6, suggesting a compatible Th1 macrophage activation profile in the latter, also consistent with previous studies [8–10].

Macrophages from mouse strains that develop a Th1 response are more readily activated to produce inflammatory mediators than those obtained from Th2 responders [4,47]. It has been shown that macrophage activation status can modulate the response to *Leishmania* infection [48–50]. Priming with IFN-γ induces the production of NO and pro-inflammatory cytokines in macrophages in response to *L*. *donovani* [35]. The expression of certain Toll-like receptors (TLRs) have been implicated in this effect [50]. We observed here that prior inflammatory conditioning with LPS allows BALB/c macrophages to develop an M1-like response, with high levels of NO and TNF. The production of these mediators enable BALB/c cells to efficiently respond to a subsequent *L*. *panamensis* challenge. Further studies are necessary to determine the mechanisms involved in the M1-like response to *L*. *panamensis* infection induced by LPS priming of BALB/c macrophages.

Taken together, our results globally suggest a classical M1 profile for activation of C57BL/6 macrophages, characterized by the induction of proinflammatory cytokines, such as TNF, as previously reported for other *Leishmania* species [8–10]. Conversely, BALB/c macrophages showed a non-classical and possibly intermediate activation profile, reflected by the simultaneous upregulation of anti-inflammatory cytokine IL-10 and M2 macrophage-secreted arginase 1, together with milder induction and/or downregulation of both inflammatory cytokines and pro-apoptotic genes. Although several studies conducted in *Leishmania*-infected BALB/c macrophages have reported downregulation of many genes involved in inflammatory processes [12–14], the occurrence of an intermediate macrophage activation profile has also been suggested [15]. Since these studies were conducted with different *L*. (*Leishmania*) species, these discrepancies could be the result of differential species-specific modulation of gene expression in macrophages [17,51]. Furthermore, variability among experimental conditions is another important factor that should be considered when interpreting the results of gene expression studies in *Leishmania*-infected murine macrophages. Differences in macrophage source (peritoneal-derived vs. bone marrow-derived), incubation time of macrophages with parasites and post-infection time points might all lead to conflicting results among studies [7]. For example, some discrepancies in the expression pattern of chemokine genes such as *Ccl3* and *Ccl4*, observed in BALB/c macrophages infected with either *L*. *donovani* or *L*. *infantum/chagasi*, may be a result of quantification at different times post-infection (96 and < 24 hours, respectively) instead of an inherent species-specific difference [12,15]. Regarding macrophage source, although baseline gene expression varies between peritoneal- and bone marrow-derived macrophages, evidence suggests that both macrophage types respond to cytokines similarly in C57BL/6 mice [8–10]. Further experiments should assess potential changes occurring at earlier post-infection time points, which will help draw a more complete picture of the effects of *L*. *panamensis* infection in these mouse strains.

Although to the best of our knowledge, this is the first study using RNA-Seq for comparing the expression patterns associated with the infection with a *Leishmania* (*Viannia*) species in C57BL/6 and BALB/c macrophages, studies in human-derived macrophages infected with *L*. *braziliensis* [52] and *L*. *panamensis* [53,54] reported mixed inflammatory and anti-inflammatory cytokine profiles. This mixed cytokine profile was associated with tissue damage, lesion chronicity and parasite persistence [52,54], and tends to resemble the profile we observed for BALB/c macrophages. This similarity further supports the use of BALB/c mice as the preferred model for studying *L*. *panamensis* immunopathogenesis and evaluating potential drug candidates [26].

## Supporting information

S1 Fig Plot of the standard deviation against the mean of raw RNA-Seq counts. Plotting the standard deviation (sd; *y* axis) against the mean (*x* axis) of raw RNA-Seq counts of each gene across samples shows that the expected variance increases with the mean.
(PDF)

S2 Fig Variance stabilizing transformation (VST) of raw RNA-Seq counts. Scatter plots of transformed counts of all pairwise comparisons of biological replicates using the VST algorithm show that variance across low- and high-count genes was reasonably compressed.
(PDF)

S3 Fig Clustered heat maps of Pearson correlation coefficients. Correlation coefficients were calculated from transformed counts to assess the similarity of RNA-Seq samples for the BALB/c **(A)** and C57BL/6 **(B)** mouse strains. The color scale is based on the correlation coefficient and was adjusted to distinguish control (uninfected) and infected samples. Clustering distance between samples was calculated using the complement of the correlation coefficient.
(PDF)

S4 Fig MA-plots of changes induced by *Leishmania panamensis* infection. The *log*_2_ fold change for infected against control samples (*y* axis) is plotted against the average of counts normalized by size factor (*x* axis) for BALB/c **(A)** and C57BL/6 **(B)** macrophages. Each gene is represented with a dot. Genes with a BH multiple-testing adjusted P value < 0.05 (highlighted in blue) were considered as differentially expressed.
(PDF)

S5 Fig Enrichment analysis performed for potentially overexpressed genes specific to the BALB/c and C57BL/6 macrophages. This analysis was performed by using global predefined GO Slim categories. Bars represent the fraction of genes from each strain clustered into each category, compared to fractions calculated for all the genes annotated in the mouse genome (reference). Number on the right side of the graph indicate the absolute value of the percent difference between the BALB/c and C57BL/6 strains.
(PDF)

S6 Fig Summary of the main findings in gene expression changes in BALB/c and C57BL/6 macrophages infected with *L*. *panamensis*. (PDF)

S1 Table Genes differentially expressed in BALB/c macrophages. (XLSX)

S2 Table Genes differentially expressed in C57BL/6 macrophages. (XLSX)

S3 Table Genes differentially expressed specific to BALB/c macrophages. (XLSX)

S4 Table Genes differentially expressed specific to C57BL/6 macrophages. (XLSX)

S5 Table Extended list of GO terms overrepresented in DE genes suspected to be reciprocally regulated in infected BALB/c and C57BL/6 macrophages. (XLSX)

S6 Table KEGG pathways enriched by DE genes of BALB/c macrophages infected with *L*. *panamensis*. (PDF)

S7 Table KEGG pathways enriched by DE genes of C57BL/6 macrophages infected with *L*. *panamensis*. (PDF)

S8 Table KEGG pathways shared by both mouse strains enriched by DE genes. (PDF)

S9 Table KEGG pathways exclusively enriched by DE in C57BL/6 macrophages infected with *L*. *panamensis*. (PDF)
