# Supplementary material for: Gene expression patterns associated with Leishmania panamensis infection in macrophages from BALB/c and C57BL/6 mice
Source: PLoS Negl Trop Dis. 2021 Feb 22;15(2):e0009225. doi: 10.1371/journal.pntd.0009225 (PMC7932533; doi:10.1371/journal.pntd.0009225)
Supplement: S1 Fig — Plotting the standard deviation (sd; y axis) against the mean (x axis) of raw RNA-Seq counts of each gene across samples shows that the expected variance increases with the mean. (PDF) [file pntd.0009225.s001.pdf]

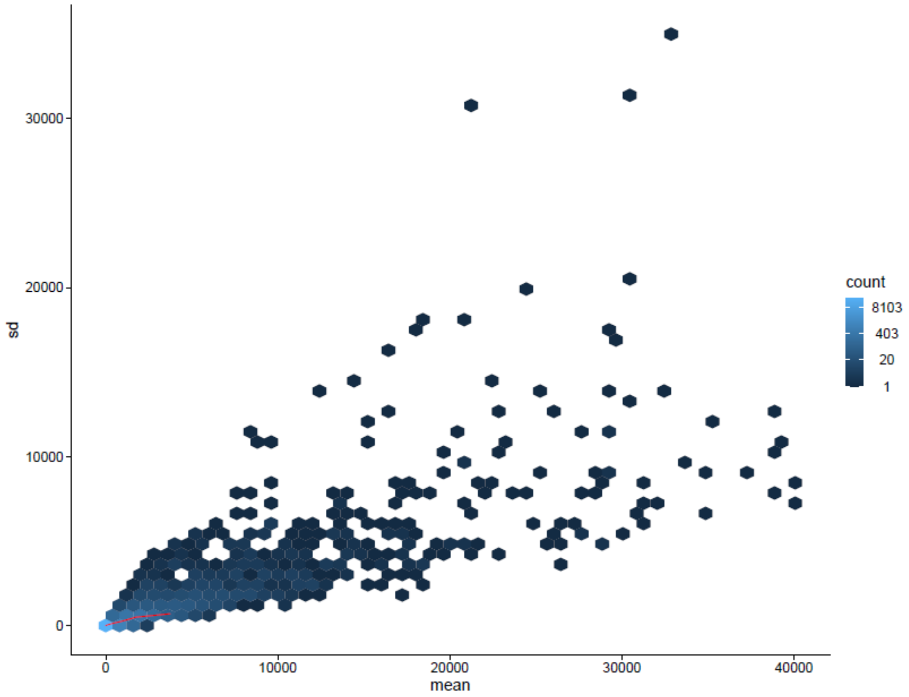

Figure S1. Plot of the standard deviation against the mean of raw RNA-Seq counts. Plotting the standard deviation (sd; *y* axis) against the mean (*x* axis) of raw RNA-Seq counts of each gene across samples shows that the expected variance increases with the mean.
