# Supplementary material for: Gene expression patterns associated with Leishmania panamensis infection in macrophages from BALB/c and C57BL/6 mice
Source: PLoS Negl Trop Dis. 2021 Feb 22;15(2):e0009225. doi: 10.1371/journal.pntd.0009225 (PMC7932533; doi:10.1371/journal.pntd.0009225)
Supplement: S2 Fig — Scatter plots of transformed counts of all pairwise comparisons of biological replicates using the VST algorithm show that variance across low- and high-count genes was reasonably compressed. (PDF) [file pntd.0009225.s002.pdf]

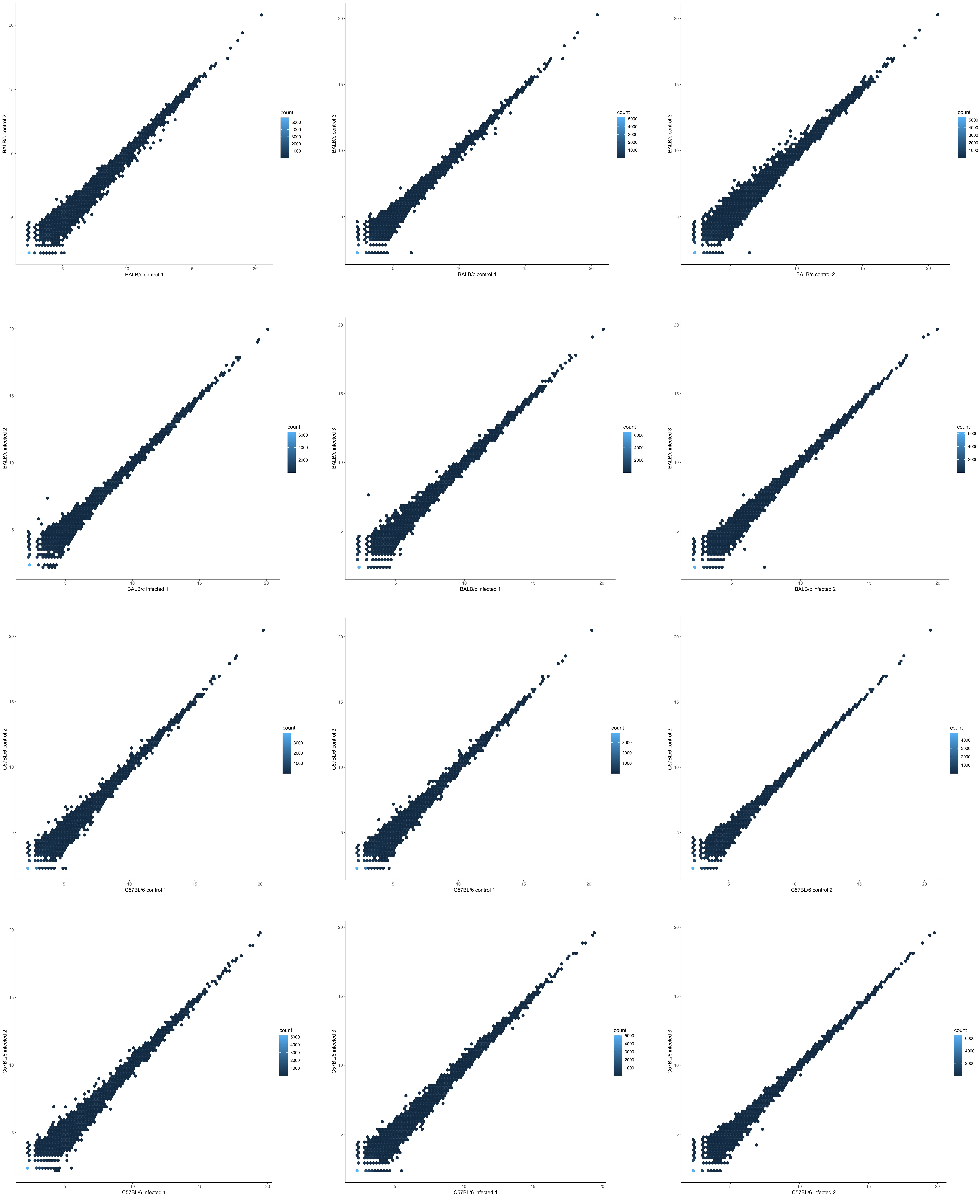

Figure S2. Variance stabilizing transformation (VST) of raw RNA-Seq counts. Scatter plots of transformed counts of all pairwise comparisons of biological replicates using the VST algorithm show that variance across low- and high-count genes was reasonably compressed.
