# Supplementary material for: Gene expression patterns associated with Leishmania panamensis infection in macrophages from BALB/c and C57BL/6 mice
Source: PLoS Negl Trop Dis. 2021 Feb 22;15(2):e0009225. doi: 10.1371/journal.pntd.0009225 (PMC7932533; doi:10.1371/journal.pntd.0009225)
Supplement: S3 Fig — Correlation coefficients were calculated from transformed counts to assess the similarity of RNA-Seq samples for the BALB/c (A) and C57BL/6 (B) mouse strains. The color scale is based on the correlation coefficient and was adjusted to distinguish control (uninfected) and infected samples. Clustering distance between samples was calculated using the complement of the correlation coefficient. (PDF) [file pntd.0009225.s003.pdf]

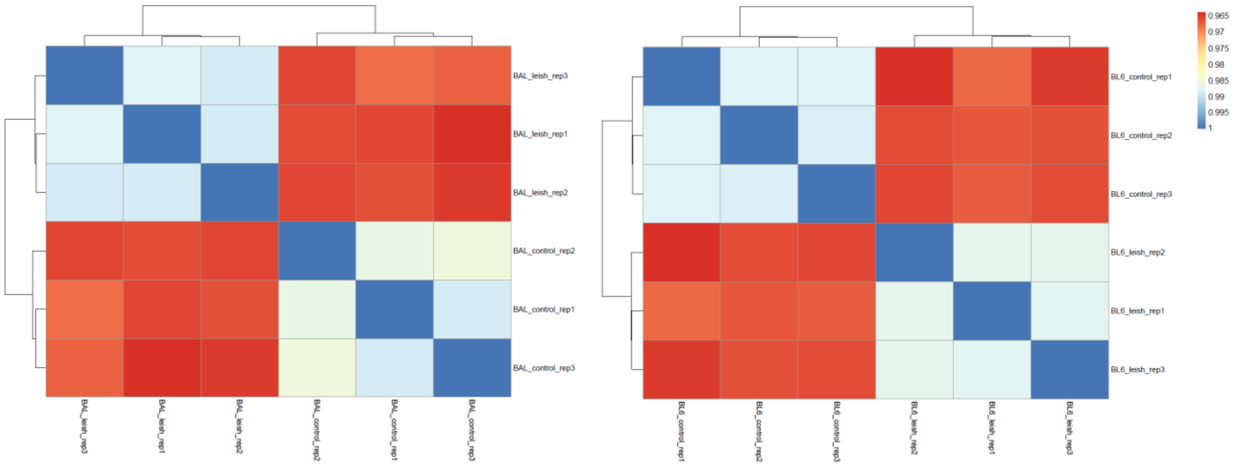

Figure S3. Clustered heat maps of Pearson correlation coefficients. Correlation coefficients were calculated from transformed counts to assess the similarity of RNA-Seq samples for the BALB/c **(A)** and C57BL/6 **(B)** mouse strains. The color scale is based on the correlation coefficient and was adjusted to distinguish control (uninfected) and infected samples. Clustering distance between samples was calculated using the complement of the correlation coefficient.
