# Supplementary material for: Gene expression patterns associated with Leishmania panamensis infection in macrophages from BALB/c and C57BL/6 mice
Source: PLoS Negl Trop Dis. 2021 Feb 22;15(2):e0009225. doi: 10.1371/journal.pntd.0009225 (PMC7932533; doi:10.1371/journal.pntd.0009225)
Supplement: S4 Fig — The log2 fold change for infected against control samples (y axis) is plotted against the average of counts normalized by size factor (x axis) for BALB/c (A) and C57BL/6 (B) macrophages. Each gene is represented with a dot. Genes with a BH multiple-testing adjusted P value < 0.05 (highlighted in blue) were considered as differentially expressed. (PDF) [file pntd.0009225.s004.pdf]

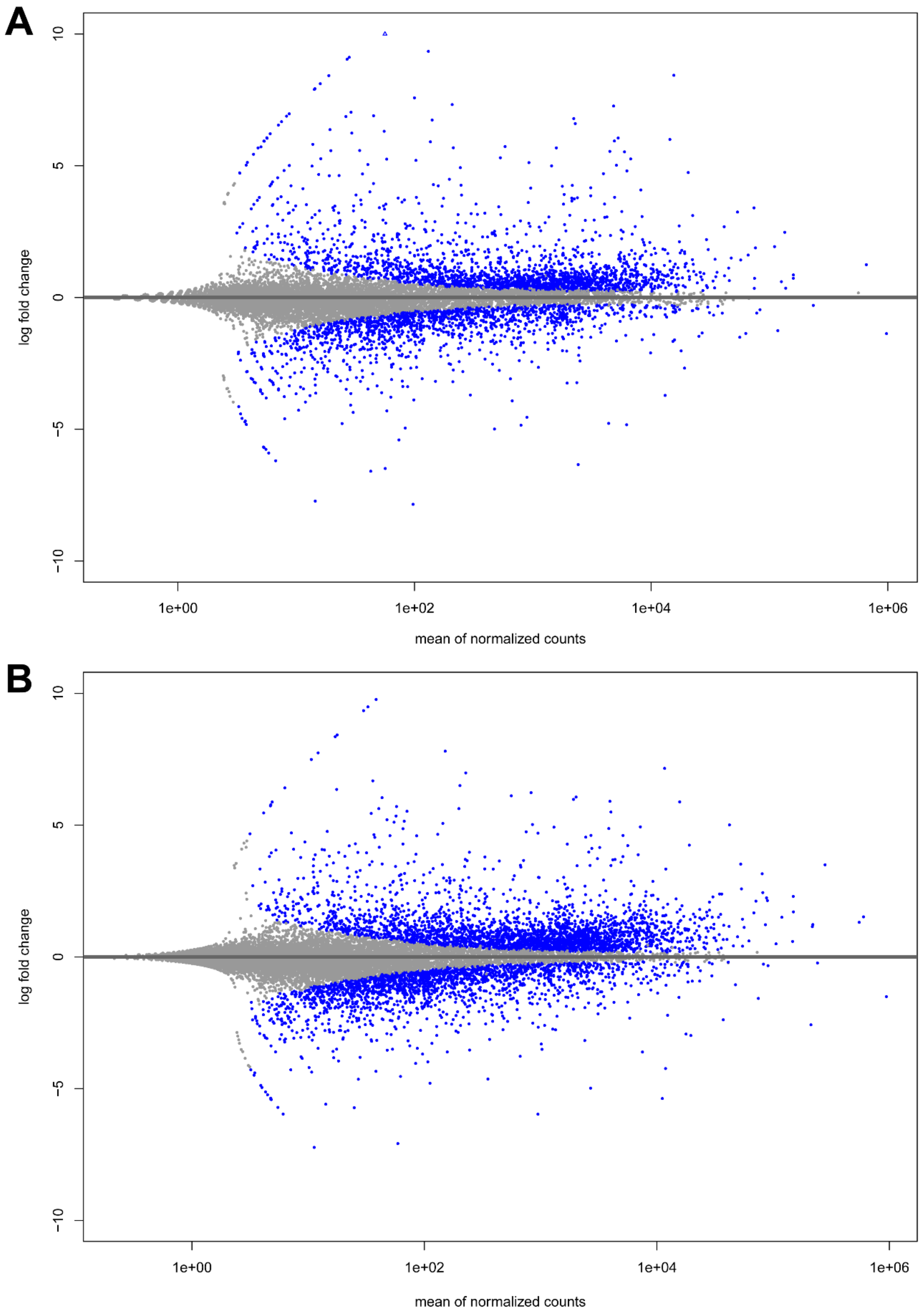

Figure S4. MA-plots of changes induced by *Leishmania panamensis* infection. The log_2 fold change for infected against control samples (*y* axis) is plotted against the average of counts normalized by size factor (*x* axis) for BALB/c **(A)** and C57BL/6 **(B)** macrophages. Each gene is represented with a dot. Genes with a BH multiple-testing adjusted P value < 0.05 (highlighted in blue) were considered as differentially expressed.
