# Supplementary material for: Gene expression patterns associated with Leishmania panamensis infection in macrophages from BALB/c and C57BL/6 mice
Source: PLoS Negl Trop Dis. 2021 Feb 22;15(2):e0009225. doi: 10.1371/journal.pntd.0009225 (PMC7932533; doi:10.1371/journal.pntd.0009225)
Supplement: S5 Fig — This analysis was performed by using global predefined GO Slim categories. Bars represent the fraction of genes from each strain clustered into each category, compared to fractions calculated for all the genes annotated in the mouse genome (reference). Number on the right side of the graph indicate the absolute value of the percent difference between the BALB/c and C57BL/6 strains. (PDF) [file pntd.0009225.s005.pdf]

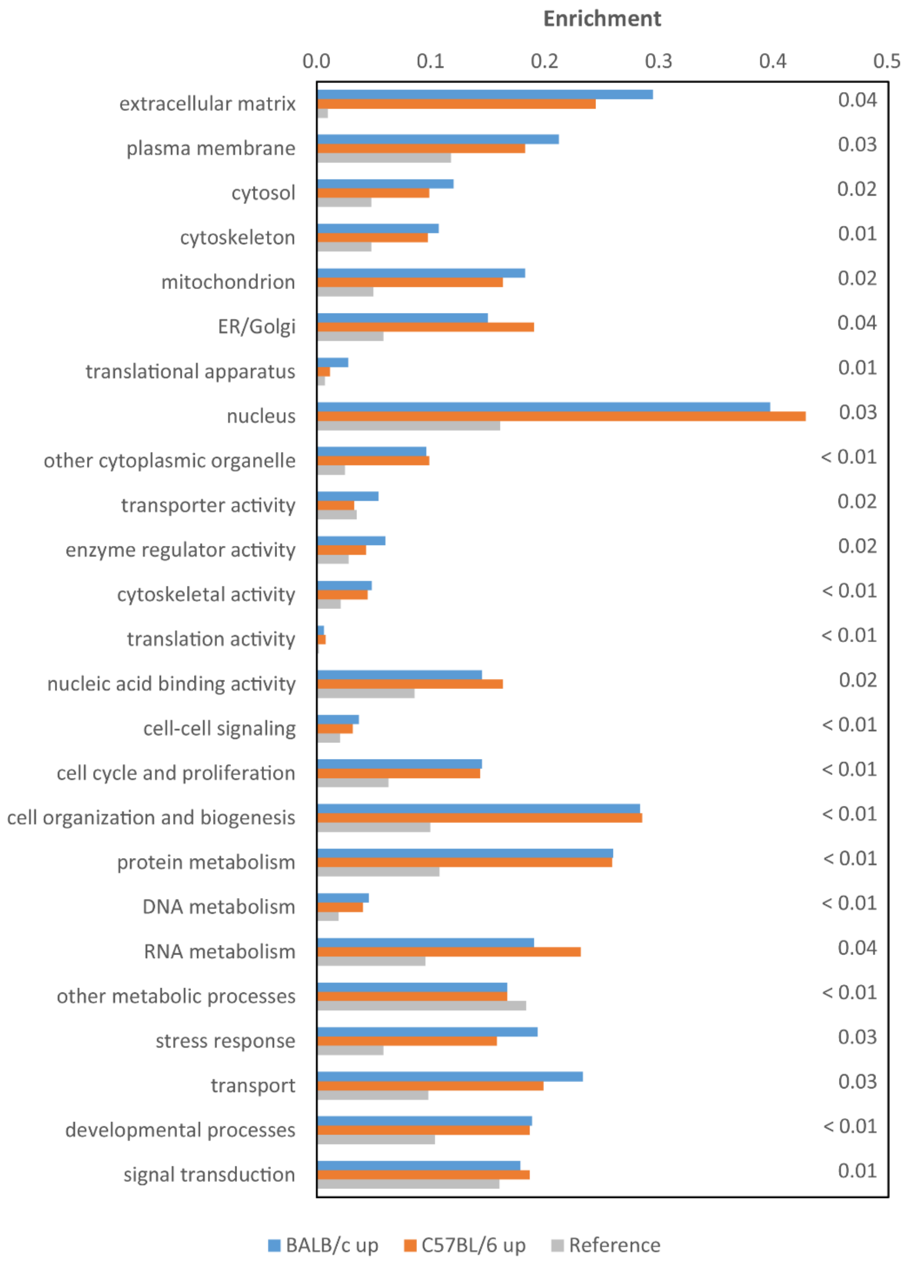

Figure S5. Enrichment analysis performed for potentially overexpressed genes specific to the BALB/c and C57BL/6 macrophages. This analysis was performed by using global predefined GO Slim categories. Bars represent the fraction of genes from each strain clustered into each category, compared to fractions calculated for all the genes annotated in the mouse genome (reference). Number on the right side of the graph indicate the absolute value of the percent difference between the BALB/c and C57BL/6 strains.
