# Supplementary material for: Gene expression patterns associated with Leishmania panamensis infection in macrophages from BALB/c and C57BL/6 mice
Source: PLoS Negl Trop Dis. 2021 Feb 22;15(2):e0009225. doi: 10.1371/journal.pntd.0009225 (PMC7932533; doi:10.1371/journal.pntd.0009225)
Supplement: S6 Fig — (PDF) [file pntd.0009225.s006.pdf]

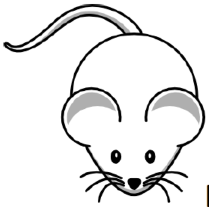

BALB/c

- Downregulation of genes associated with lysosomal degradation.
- Weak induction of chemokine genes.
- Non-induction of key regulators MAPK11/p38 MAPK-β and MAP3K5/ASK1.
- Upregulation of the anti-inflammatory cytokine IL-10.
- Moderate upregulation of arginase 1 and upregulation of the gene enconding its transporter CAT2.
- General activation of genes involved in regulation of cell cycle and apoptosis but showing downregulation or moderate expression of some key regulators
- Gene expression pattern consistent with a intermediate inflammatory response.

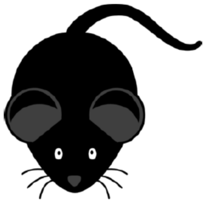

C57BL/6

- Upregulation of genes associated with lysosomal degradation.
- Consistent induction of chemokine genes for recruitment of monocytes and neutrophils.
- Upregulation of genes encoding MAPK genes known for regulating the activation of inflammatory cytokines and apoptotic signals.
- Downregulation of the anti-inflammatory cytokine IL-10.
- High expression of the gene encoding arginase 1 but no change in expression of the gene encoding its transporter CAT2.
- Strong induction of genes involved in regulation of cell cycle and apoptosis.
- Gene expression pattern associated with a classic macrophage M1 activation.

Figure S6. Summary of the main findings in gene expression changes in BALB/c and C57BL/6 macrophages infected with *L. panamensis*. Mice drawings were downloaded as free clipart from Clker.com (https://www.clker.com/).
