# Supplementary material for: Gene expression patterns associated with Leishmania panamensis infection in macrophages from BALB/c and C57BL/6 mice
Source: PLoS Negl Trop Dis. 2021 Feb 22;15(2):e0009225. doi: 10.1371/journal.pntd.0009225 (PMC7932533; doi:10.1371/journal.pntd.0009225)
Supplement: S6 Table — (PDF) [file pntd.0009225.s012.pdf]

Table S6. KEGG pathways enriched by DE genes of BALB/c macrophages infected with *L. panamensis.*

Accession number	KEGG Pathway	No. of DE genes	Pathway size	Adjusted *P* value
**Enriched by upregulated genes**				
mmu05012	Parkinson disease	94	247	1.45E-29
mmu03050	Proteasome	34	47	8.40E-22
mmu00190	Oxidative phosphorylation	58	133	9.95E-22
mmu05016	Huntington disease	93	303	9.95E-22
mmu05010	Alzheimer disease	90	368	5.98E-14
mmu04145	Phagosome	54	180	5.93E-12
mmu05169	Epstein-Barr virus infection	62	228	1.06E-11
mmu04714	Thermogenesis	56	230	1.53E-08
mmu04612	Antigen processing and presentation	30	91	1.04E-07
mmu04142	Lysosome	37	131	1.78E-07
mmu05323	Rheumatoid arthritis	27	86	1.69E-06
mmu05418	Fluid shear stress and atherosclerosis	37	144	2.13E-06
mmu04932	Non-alcoholic fatty liver disease (NAFLD)	38	150	2.13E-06
mmu05203	Viral carcinogenesis	50	230	3.93E-06
mmu05164	Influenza A	39	166	1.06E-05
mmu04966	Collecting duct acid secretion	13	27	1.28E-05
mmu05132	Salmonella infection	45	220	7.71E-05
mmu04144	Endocytosis	52	270	8.58E-05
mmu05017	Spinocerebellar ataxia	31	131	1.04E-04
mmu04141	Protein processing in endoplasmic reticulum	36	164	1.15E-04
mmu03060	Protein export	12	28	1.21E-04
mmu04210	Apoptosis	31	136	2.02E-04
mmu05167	Kaposi sarcoma-associated herpesvirus infection	43	218	2.40E-04
mmu05330	Allograft rejection	18	63	5.19E-04
mmu05160	Hepatitis C	33	160	8.29E-04
mmu05134	Legionellosis	17	61	1.08E-03
mmu05163	Human cytomegalovirus infection	46	255	1.08E-03
mmu00480	Glutathione metabolism	18	67	1.08E-03
mmu04218	Cellular senescence	36	185	1.17E-03
mmu03030	DNA replication	12	35	1.17E-03
mmu04217	Necroptosis	34	173	1.42E-03
mmu05170	Human immunodeficiency virus 1 infection	43	238	1.48E-03
mmu00020	Citrate cycle (TCA cycle)	11	32	1.97E-03
mmu04721	Synaptic vesicle cycle	19	77	1.97E-03
mmu03040	Spliceosome	30	150	2.22E-03
mmu05165	Human papillomavirus infection	58	361	2.88E-03

mmu01200	Carbon metabolism	25	120	3.53E-03
mmu04061	Viral protein interaction with cytokine and cytokine receptor	22	102	4.43E-03
mmu04940	Type I diabetes mellitus	17	70	4.43E-03
mmu04110	Cell cycle	25	123	4.81E-03
mmu05162	Measles	28	144	4.82E-03
mmu05332	Graft-versus-host disease	16	65	5.06E-03
mmu05166	Human T-cell leukemia virus 1 infection	41	246	8.47E-03
mmu05416	Viral myocarditis	19	88	8.99E-03
mmu04621	NOD-like receptor signaling pathway	36	213	1.23E-02
mmu01210	2-Oxocarboxylic acid metabolism	7	19	1.31E-02
mmu05320	Autoimmune thyroid disease	17	78	1.32E-02
mmu04114	Oocyte meiosis	23	119	1.32E-02
mmu04979	Cholesterol metabolism	12	49	2.05E-02
mmu03420	Nucleotide excision repair	11	43	2.05E-02
mmu04064	NF-kappa B signaling pathway	21	110	2.21E-02
mmu01230	Biosynthesis of amino acids	16	77	2.67E-02
mmu00010	Glycolysis / Gluconeogenesis	14	66	3.67E-02
mmu03013	RNA transport	31	191	3.79E-02
mmu00062	Fatty acid elongation	8	29	4.01E-02
mmu05142	Chagas disease (American trypanosomiasis)	19	102	4.01E-02
**Enriched by downregulated genes**				
mmu04658	Th1 and Th2 cell differentiation	17	87	1.01E-03
mmu00100	Steroid biosynthesis	8	20	1.01E-03
mmu04062	Chemokine signaling pathway	27	196	1.04E-03
mmu04672	Intestinal immune network for IgA production	11	42	1.04E-03
mmu05152	Tuberculosis	25	178	1.04E-03
mmu05321	Inflammatory bowel disease (IBD)	13	60	1.04E-03
mmu05140	Leishmaniasis	14	69	1.04E-03
mmu04625	C-type lectin receptor signaling pathway	18	112	1.86E-03
mmu04640	Hematopoietic cell lineage	16	95	2.42E-03
mmu04610	Complement and coagulation cascades	15	93	6.00E-03
mmu05145	Toxoplasmosis	15	108	2.46E-02
mmu05220	Chronic myeloid leukemia	12	76	2.46E-02
mmu04068	FoxO signaling pathway	17	131	2.46E-02
mmu04064	NF-kappa B signaling pathway	15	110	2.67E-02
mmu05224	Breast cancer	18	147	2.80E-02
mmu04668	TNF signaling pathway	15	113	2.80E-02
mmu04061	Viral protein interaction with cytokine and cytokine receptor	14	102	2.80E-02
mmu05142	Chagas disease (American trypanosomiasis)	14	102	2.80E-02
mmu05226	Gastric cancer	18	150	3.01E-02
