# Supplementary material for: Gene expression patterns associated with Leishmania panamensis infection in macrophages from BALB/c and C57BL/6 mice
Source: PLoS Negl Trop Dis. 2021 Feb 22;15(2):e0009225. doi: 10.1371/journal.pntd.0009225 (PMC7932533; doi:10.1371/journal.pntd.0009225)
Supplement: S7 Table — (PDF) [file pntd.0009225.s013.pdf]

Table S7. KEGG pathways enriched by DE genes of C57BL/6 macrophages infected with *L. panamensis.*

Accession number	KEGG Pathway	No. of DE genes	Pathway size	Adjusted *P* value
**Enriched by upregulated genes**				
mmu05012	Parkinson disease	108	247	4.78E-30
mmu00190	Oxidative phosphorylation	72	133	4.91E-27
mmu05016	Huntington disease	112	303	5.60E-24
mmu03050	Proteasome	34	47	2.76E-18
mmu05010	Alzheimer disease	111	368	5.58E-16
mmu04714	Thermogenesis	74	230	4.70E-12
mmu04142	Lysosome	46	131	8.26E-09
mmu04932	Non-alcoholic fatty liver disease (NAFLD)	50	150	9.85E-09
mmu05169	Epstein-Barr virus infection	65	228	3.52E-08
mmu04145	Phagosome	55	180	4.02E-08
mmu05132	Salmonella infection	60	220	7.86E-07
mmu05203	Viral carcinogenesis	61	230	1.68E-06
mmu05418	Fluid shear stress and atherosclerosis	43	144	4.05E-06
mmu04141	Protein processing in endoplasmic reticulum	47	164	4.05E-06
mmu03020	RNA polymerase	16	30	4.78E-06
mmu05323	Rheumatoid arthritis	30	86	6.28E-06
mmu04612	Antigen processing and presentation	31	91	6.91E-06
mmu03060	Protein export	15	28	8.88E-06
mmu00480	Glutathione metabolism	25	67	1.19E-05
mmu03040	Spliceosome	42	150	2.49E-05
mmu05164	Influenza A	45	166	2.70E-05
mmu04144	Endocytosis	64	270	3.38E-05
mmu04217	Necroptosis	46	173	3.39E-05
mmu01200	Carbon metabolism	35	120	5.59E-05
mmu00020	Citrate cycle (TCA cycle)	15	32	5.70E-05
mmu05163	Human cytomegalovirus infection	59	255	1.54E-04
mmu04210	Apoptosis	37	136	1.54E-04
mmu04966	Collecting duct acid secretion	13	27	1.54E-04
mmu05134	Legionellosis	21	61	2.54E-04
mmu05167	Kaposi sarcoma-associated herpesvirus infection	51	218	3.79E-04
mmu05160	Hepatitis C	40	160	5.28E-04
mmu04668	TNF signaling pathway	31	113	5.44E-04
mmu03013	RNA transport	45	191	8.13E-04
mmu00010	Glycolysis / Gluconeogenesis	21	66	8.13E-04
mmu00052	Galactose metabolism	13	32	1.12E-03
mmu01210	2-Oxocarboxylic acid metabolism	9	19	3.07E-03
mmu00330	Arginine and proline metabolism	17	53	3.07E-03

mmu04114	Oocyte meiosis	30	119	3.07E-03
mmu05017	Spinocerebellar ataxia	32	131	3.56E-03
mmu04623	Cytosolic DNA-sensing pathway	19	64	3.99E-03
mmu04218	Cellular senescence	41	185	5.16E-03
mmu05170	Human immunodeficiency virus 1 infection	50	238	5.16E-03
mmu00062	Fatty acid elongation	11	29	6.02E-03
mmu00270	Cysteine and methionine metabolism	16	52	6.41E-03
mmu01230	Biosynthesis of amino acids	21	77	6.41E-03
mmu04979	Cholesterol metabolism	15	49	9.12E-03
mmu03030	DNA replication	12	35	9.12E-03
mmu04061	Viral protein interaction with cytokine and cytokine receptor	25	102	1.11E-02
mmu05034	Alcoholism	42	201	1.28E-02
mmu05330	Allograft rejection	17	63	1.94E-02
mmu03420	Nucleotide excision repair	13	43	1.95E-02
mmu05142	Chagas disease (American trypanosomiasis)	24	102	2.23E-02
mmu04723	Retrograde endocannabinoid signaling	32	148	2.23E-02
mmu00520	Amino sugar and nucleotide sugar metabolism	14	49	2.31E-02
mmu04940	Type I diabetes mellitus	18	70	2.38E-02
mmu05165	Human papillomavirus infection	66	361	2.66E-02
mmu04064	NF-kappa B signaling pathway	25	110	2.76E-02
mmu00983	Drug metabolism - other enzymes	21	88	2.86E-02
mmu04721	Synaptic vesicle cycle	19	77	2.86E-02
mmu04621	NOD-like receptor signaling pathway	42	213	3.13E-02
mmu04120	Ubiquitin mediated proteolysis	30	143	3.96E-02
mmu04657	IL-17 signaling pathway	21	91	4.07E-02
mmu05162	Measles	30	144	4.24E-02
mmu00500	Starch and sucrose metabolism	10	33	4.42E-02
mmu00620	Pyruvate metabolism	11	38	4.42E-02
mmu04260	Cardiac muscle contraction	20	87	4.74E-02
mmu05332	Graft-versus-host disease	16	65	4.86E-02
**Enriched by downregulated genes**				
mmu05140	Leishmaniasis	24	69	1.66E-09
mmu05152	Tuberculosis	39	178	3.43E-09
mmu04658	Th1 and Th2 cell differentiation	24	87	1.31E-07
mmu04640	Hematopoietic cell lineage	22	95	1.50E-05
mmu05145	Toxoplasmosis	23	108	3.18E-05
mmu04064	NF-kappa B signaling pathway	23	110	3.74E-05
mmu05321	Inflammatory bowel disease (IBD)	16	60	5.35E-05
mmu04625	C-type lectin receptor signaling pathway	22	112	1.46E-04
mmu04659	Th17 cell differentiation	20	102	3.29E-04

mmu05142	Chagas disease (American trypanosomiasis)	20	102	3.29E-04
mmu04672	Intestinal immune network for IgA production	12	42	3.39E-04
mmu05168	Herpes simplex virus 1 infection	53	437	3.65E-04
mmu04662	B cell receptor signaling pathway	17	81	4.54E-04
mmu05143	African trypanosomiasis	11	38	5.36E-04
mmu04933	AGE-RAGE signaling pathway in diabetic complications	19	101	6.68E-04
mmu04668	TNF signaling pathway	20	113	9.78E-04
mmu05235	PD-L1 expression and PD-1 checkpoint pathway in cancer	17	88	1.03E-03
mmu05144	Malaria	13	56	1.03E-03
mmu05161	Hepatitis B	25	162	1.10E-03
mmu04062	Chemokine signaling pathway	28	196	1.54E-03
mmu05310	Asthma	8	24	1.54E-03
mmu05169	Epstein-Barr virus infection	31	228	1.60E-03
mmu00100	Steroid biosynthesis	7	20	2.71E-03
mmu05164	Influenza A	24	166	3.36E-03
mmu05224	Breast cancer	22	147	3.51E-03
mmu04068	FoxO signaling pathway	20	131	4.62E-03
mmu04660	T cell receptor signaling pathway	17	103	4.62E-03
mmu05220	Chronic myeloid leukemia	14	76	4.62E-03
mmu05150	Staphylococcus aureus infection	19	122	4.62E-03
mmu05166	Human T-cell leukemia virus 1 infection	31	246	4.62E-03
mmu05162	Measles	21	144	5.49E-03
mmu04620	Toll-like receptor signaling pathway	16	99	7.67E-03
mmu04931	Insulin resistance	17	110	8.72E-03
mmu05223	Non-small cell lung cancer	12	66	1.11E-02
mmu05133	Pertussis	13	76	1.24E-02
mmu04145	Phagosome	23	180	1.67E-02
mmu04380	Osteoclast differentiation	18	128	1.69E-02
mmu05215	Prostate cancer	15	99	1.79E-02
mmu05226	Gastric cancer	20	150	1.79E-02
mmu04211	Longevity regulating pathway	14	90	1.84E-02
mmu05202	Transcriptional misregulation in cancer	23	184	1.96E-02
mmu04722	Neurotrophin signaling pathway	17	121	1.97E-02
mmu05222	Small cell lung cancer	14	92	2.10E-02
mmu04061	Viral protein interaction with cytokine and cytokine receptor	15	102	2.10E-02
mmu01522	Endocrine resistance	14	93	2.19E-02
mmu04610	Complement and coagulation cascades	14	93	2.19E-02
mmu05323	Rheumatoid arthritis	13	86	2.79E-02
mmu01040	Biosynthesis of unsaturated fatty acids	7	32	2.79E-02
mmu05225	Hepatocellular carcinoma	21	172	3.20E-02

mmu01524	Platinum drug resistance	12	78	3.20E-02
mmu04919	Thyroid hormone signaling pathway	16	120	3.58E-02
mmu05020	Prion diseases	7	34	3.66E-02
mmu01212	Fatty acid metabolism	10	61	3.75E-02
mmu05134	Legionellosis	10	61	3.75E-02
mmu04060	Cytokine-cytokine receptor interaction	31	295	4.39E-02
