# Supplementary material for: Gene expression patterns associated with Leishmania panamensis infection in macrophages from BALB/c and C57BL/6 mice
Source: PLoS Negl Trop Dis. 2021 Feb 22;15(2):e0009225. doi: 10.1371/journal.pntd.0009225 (PMC7932533; doi:10.1371/journal.pntd.0009225)
Supplement: S8 Table — (PDF) [file pntd.0009225.s014.pdf]

Table S8. KEGG pathways shared by both mouse strains enriched by DE genes.

Accession number	KEGG Pathway	No. of DE genes	Pathway size	Adjusted *P* value
**Enriched by upregulated genes**				
mmu05012	Parkinson disease	108	247	4.78E-30
mmu00190	Oxidative phosphorylation	72	133	4.91E-27
mmu05016	Huntington disease	112	303	5.60E-24
mmu03050	Proteasome	34	47	2.76E-18
mmu05010	Alzheimer disease	111	368	5.58E-16
mmu04714	Thermogenesis	74	230	4.70E-12
mmu04142	Lysosome	46	131	8.26E-09
mmu04932	Non-alcoholic fatty liver disease (NAFLD)	50	150	9.85E-09
mmu05169	Epstein-Barr virus infection	65	228	3.52E-08
mmu04145	Phagosome	55	180	4.02E-08
mmu05132	Salmonella infection	60	220	7.86E-07
mmu05203	Viral carcinogenesis	61	230	1.68E-06
mmu05418	Fluid shear stress and atherosclerosis	43	144	4.05E-06
mmu04141	Protein processing in endoplasmic reticulum	47	164	4.05E-06
mmu05323	Rheumatoid arthritis	30	86	6.28E-06
mmu04612	Antigen processing and presentation	31	91	6.91E-06
mmu03060	Protein export	15	28	8.88E-06
mmu00480	Glutathione metabolism	25	67	1.19E-05
mmu03040	Spliceosome	42	150	2.49E-05
mmu05164	Influenza A	45	166	2.70E-05
mmu04144	Endocytosis	64	270	3.38E-05
mmu04217	Necroptosis	46	173	3.39E-05
mmu01200	Carbon metabolism	35	120	5.59E-05
mmu00020	Citrate cycle (TCA cycle)	15	32	5.70E-05
mmu05163	Human cytomegalovirus infection	59	255	1.54E-04
mmu04210	Apoptosis	37	136	1.54E-04
mmu04966	Collecting duct acid secretion	13	27	1.54E-04
mmu05134	Legionellosis	21	61	2.54E-04
mmu05167	Kaposi sarcoma-associated herpesvirus infection	51	218	3.79E-04
mmu05160	Hepatitis C	40	160	5.28E-04
mmu03013	RNA transport	45	191	8.13E-04
mmu00010	Glycolysis / Gluconeogenesis	21	66	8.13E-04
mmu01210	2-Oxocarboxylic acid metabolism	9	19	3.07E-03
mmu04114	Oocyte meiosis	30	119	3.07E-03
mmu05017	Spinocerebellar ataxia	32	131	3.56E-03
mmu04218	Cellular senescence	41	185	5.16E-03
mmu05170	Human immunodeficiency virus 1 infection	50	238	5.16E-03

mmu00062	Fatty acid elongation	11	29	6.02E-03
mmu01230	Biosynthesis of amino acids	21	77	6.41E-03
mmu04979	Cholesterol metabolism	15	49	9.12E-03
mmu03030	DNA replication	12	35	9.12E-03
mmu04061	Viral protein interaction with cytokine and cytokine receptor	25	102	1.11E-02
mmu05330	Allograft rejection	17	63	1.94E-02
mmu03420	Nucleotide excision repair	13	43	1.95E-02
mmu05142	Chagas disease (American trypanosomiasis)	24	102	2.23E-02
mmu04940	Type I diabetes mellitus	18	70	2.38E-02
mmu05165	Human papillomavirus infection	66	361	2.66E-02
mmu04064	NF-kappa B signaling pathway	25	110	2.76E-02
mmu04721	Synaptic vesicle cycle	19	77	2.86E-02
mmu04621	NOD-like receptor signaling pathway	42	213	3.13E-02
mmu05162	Measles	30	144	4.24E-02
mmu05332	Graft-versus-host disease	16	65	4.86E-02
**Enriched by downregulated genes**				
mmu05140	Leishmaniasis	24	69	1.66E-09
mmu05152	Tuberculosis	39	178	3.43E-09
mmu04658	Th1 and Th2 cell differentiation	24	87	1.31E-07
mmu04640	Hematopoietic cell lineage	22	95	1.50E-05
mmu05145	Toxoplasmosis	23	108	3.18E-05
mmu04064	NF-kappa B signaling pathway	23	110	3.74E-05
mmu05321	Inflammatory bowel disease (IBD)	16	60	5.35E-05
mmu04625	C-type lectin receptor signaling pathway	22	112	1.46E-04
mmu05142	Chagas disease (American trypanosomiasis)	20	102	3.29E-04
mmu04672	Intestinal immune network for IgA production	12	42	3.39E-04
mmu04668	TNF signaling pathway	20	113	9.78E-04
mmu04062	Chemokine signaling pathway	28	196	1.54E-03
mmu00100	Steroid biosynthesis	7	20	2.71E-03
mmu05224	Breast cancer	22	147	3.51E-03
mmu04068	FoxO signaling pathway	20	131	4.62E-03
mmu05220	Chronic myeloid leukemia	14	76	4.62E-03
mmu05226	Gastric cancer	20	150	1.79E-02
mmu04061	Viral protein interaction with cytokine and cytokine receptor	15	102	2.10E-02
mmu04610	Complement and coagulation cascades	14	93	2.19E-02
