# Supplementary material for: Gene expression patterns associated with Leishmania panamensis infection in macrophages from BALB/c and C57BL/6 mice
Source: PLoS Negl Trop Dis. 2021 Feb 22;15(2):e0009225. doi: 10.1371/journal.pntd.0009225 (PMC7932533; doi:10.1371/journal.pntd.0009225)
Supplement: S9 Table — (PDF) [file pntd.0009225.s015.pdf]

Table S9. KEGG pathways exclusively enriched by DE genes in C57BL/6 macrophages infected with *L. panamensis.*

Accession number [1]	KEGG Pathway	DE genes	Pathway size	Adjusted *P* value
**Enriched by upregulated genes**				
mmu03020	RNA polymerase	16	30	4.78E-06
mmu04668	TNF signaling pathway	31	113	5.44E-04
mmu00052	Galactose metabolism	13	32	1.12E-03
mmu00330	Arginine and proline metabolism	17	53	3.07E-03
mmu04623	Cytosolic DNA-sensing pathway	19	64	3.99E-03
mmu00270	Cysteine and methionine metabolism	16	52	6.41E-03
mmu05034	Alcoholism	42	201	1.28E-02
mmu04723	Retrograde endocannabinoid signaling	32	148	2.23E-02
mmu00520	Amino- and nucleotide-sugar metabolism	14	49	2.31E-02
mmu00983	Drug metabolism - other enzymes	21	88	2.86E-02
mmu04120	Ubiquitin mediated proteolysis	30	143	3.96E-02
mmu04657	IL-17 signaling pathway	21	91	4.07E-02
mmu00500	Starch and sucrose metabolism	10	33	4.42E-02
mmu00620	Pyruvate metabolism	11	38	4.42E-02
mmu04260	Cardiac muscle contraction	20	87	4.74E-02
**Enriched by downregulated genes**				
mmu04659	Th17 cell differentiation	20	102	3.29E-04
mmu05168	Herpes simplex virus 1 infection	53	437	3.65E-04
mmu04662	B cell receptor signaling pathway	17	81	4.54E-04
mmu05143	African trypanosomiasis	11	38	5.36E-04
mmu04933	AGE-RAGE signaling pathway in diabetic complications	19	101	6.68E-04
mmu05235	PD-L1 expression and PD-1 checkpoint pathway in cancer	17	88	1.03E-03
mmu05144	Malaria	13	56	1.03E-03
mmu05161	Hepatitis B	25	162	1.10E-03
mmu05310	Asthma	8	24	1.54E-03
mmu05169	Epstein-Barr virus infection	31	228	1.60E-03
mmu05164	Influenza A	24	166	3.36E-03
mmu04660	T cell receptor signaling pathway	17	103	4.62E-03
mmu05150	Staphylococcus aureus infection	19	122	4.62E-03
mmu05166	Human T-cell leukemia virus 1 infection	31	246	4.62E-03
mmu05162	Measles	21	144	5.49E-03
mmu04620	Toll-like receptor signaling pathway	16	99	7.67E-03
mmu04931	Insulin resistance	17	110	8.72E-03
mmu05223	Non-small cell lung cancer	12	66	1.11E-02
mmu05133	Pertussis	13	76	1.24E-02
mmu04145	Phagosome	23	180	1.67E-02

mmu04380	Osteoclast differentiation	18	128	1.69E-02
mmu05215	Prostate cancer	15	99	1.79E-02
mmu04211	Longevity regulating pathway	14	90	1.84E-02
mmu05202	Transcriptional misregulation in cancer	23	184	1.96E-02
mmu04722	Neurotrophin signaling pathway	17	121	1.97E-02
mmu05222	Small cell lung cancer	14	92	2.10E-02
mmu01522	Endocrine resistance	14	93	2.19E-02
mmu05323	Rheumatoid arthritis	13	86	2.79E-02
mmu01040	Biosynthesis of unsaturated fatty acids	7	32	2.79E-02
mmu05225	Hepatocellular carcinoma	21	172	3.20E-02
mmu01524	Platinum drug resistance	12	78	3.20E-02
mmu04919	Thyroid hormone signaling pathway	16	120	3.58E-02
mmu05020	Prion diseases	7	34	3.66E-02
mmu01212	Fatty acid metabolism	10	61	3.75E-02
mmu05134	Legionellosis	10	61	3.75E-02
mmu04060	Cytokine-cytokine receptor interaction	31	295	4.39E-02
